# Global siRNA screen identifies human host factors critical for SARS-CoV-2 replication and late stages of infection

**DOI:** 10.1371/journal.pbio.3002738

**Published:** 2025-06-12

**Authors:** Xin Yin, Yuan Pu, Shuofeng Yuan, Lars Pache, Christopher Churas, Stuart Weston, Laura Riva, Lacy M. Simons, William J. Cisneros, Thomas Clausen, Grace Biddle, Simon Doss-Gollin, Meagan Deming, Paul D. De Jesus, Ha Na Kim, Daniel Fuentes, John M. Whitelock, Jeffrey D. Esko, Megan S. Lord, Ignacio Mena, Adolfo García-Sastre, Judd F. Hultquist, Matthew B. Frieman, Trey Ideker, Dexter Pratt, Laura Martin-Sancho, Sumit K. Chanda

**Affiliations:** 1 State Key Laboratory for Animal Disease Control and Prevention, Harbin Veterinary Research Institute, Chinese Academy of Agricultural Sciences, Harbin, China; 2 Department of Immunology and Microbiology, The Scripps Research Institute, La Jolla, California, United States of America; 3 Department of Microbiology, Li Ka Shing Faculty of Medicine, The University of Hong Kong, Pokfulam, Hong Kong SAR, China,; 4 NCI Designated Cancer Center, Sanford Burnham Prebys Medical Discovery Institute, La Jolla, California, United States of America; 5 Department of Medicine, University of California San Diego, La Jolla, California, United States of America; 6 Department of Microbiology and Immunology, University of Maryland School of Medicine, Baltimore, Maryland, United States of America; 7 Calibr-Skaggs at Scripps Research Institute, La Jolla, California, United States of America; 8 Division of Infectious Diseases, Departments of Medicine and Microbiology-Immunology, Northwestern University Feinberg School of Medicine, Chicago, Illinois, United States of America; 9 Department of Cellular and Molecular Medicine, University of California, San Diego, La Jolla, California, United States of America; 10 Graduate School of Biomedical Engineering, University of New South Wales, Sydney, Australia; 11 Department of Microbiology, Icahn School of Medicine at Mount Sinai, New York, New York, United States of America; 12 Global Health and Emerging Pathogens Institute, Icahn School of Medicine at Mount Sinai, New York, New York, United States of America; 13 Department of Medicine, Division of Infectious Diseases, Icahn School of Medicine at Mount Sinai, New York, New York, United States of America; 14 The Tisch Institute, Icahn School of Medicine at Mount Sinai, New York, New York, United States of America; 15 Department of Pathology, Molecular and Cell-Based Medicine, Icahn School of Medicine at Mount Sinai, New York, New York, United States of America; 16 The Icahn Genomics Institute, Icahn School of Medicine at Mount Sinai, New York, New York, United States of America; 17 Department of Computer Science and Engineering, University of California San Diego, La Jolla, California, United States of America; 18 Department of Infectious Disease, Imperial College London, London, United Kingdom; Ulm University Medical Center, GERMANY

## Abstract

Defining the subset of cellular factors governing SARS-CoV-2 replication can provide critical insights into viral pathogenesis and identify targets for host-directed antiviral therapies. While a number of genetic screens have previously reported SARS-CoV-2 host dependency factors, most of these approaches relied on utilizing pooled genome-scale CRISPR libraries, which are biased toward the discovery of host proteins impacting early stages of viral replication. To identify host factors involved throughout the SARS-CoV-2 infectious cycle, we conducted an arrayed genome-scale siRNA screen. Resulting data were integrated with published functional screens and proteomics data to reveal (i) common pathways that were identified in all OMICs datasets—including regulation of Wnt signaling and gap junctions, (ii) pathways uniquely identified in this screen—including NADH oxidation, or (iii) pathways supported by this screen and proteomics data but not published functional screens—including arachionate production and MAPK signaling. The identified proviral host factors were mapped into the SARS-CoV-2 infectious cycle, including 32 proteins that were determined to impact viral replication and 27 impacting late stages of infection, respectively. Additionally, a subset of proteins was tested across other coronaviruses revealing a subset of proviral factors that were conserved across pandemic SARS-CoV-2, epidemic SARS-CoV-1 and MERS-CoV, and the seasonal coronavirus OC43-CoV. Further studies illuminated a role for the heparan sulfate proteoglycan perlecan in SARS-CoV-2 viral entry and found that inhibition of the non-canonical NF-kB pathway through targeting of BIRC2 restricts SARS-CoV-2 replication both in vitro and in vivo. These studies provide critical insight into the landscape of virus–host interactions driving SARS-CoV-2 replication as well as valuable targets for host-directed antivirals.

## Abstract 2

Most genetic screens characterize host factors important for the early stages of viral infection. This study uses a genome-scale siRNA screen designed to uncover key pathways influencing both early and late stages of infection, including the non-canonical NF-κB pathway.

## Introduction

As of February 2025, severe acute respiratory syndrome coronavirus 2 (SARS-CoV-2), the causative agent of COVID-19, has infected more than 777 million people worldwide and led to over 7 million deaths according to the World Health Organization (WHO). In the last 21 years, other coronaviruses have caused zoonotic outbreaks of severe viral respiratory illness in the human population. These include SARS-CoV-1, which was first reported in 2003 and has caused over 8,000 infections with a mortality rate of 9.5% [1], and MERS, which was initially reported in 2012 and is responsible for over 2,500 infections with a 34.4% fatality rate [2]. Four years after the SARS-CoV-2 pandemic was declared, and despite available therapeutics and vaccines, the virus still remains a global health threat due to vaccine hesitancy, limited rollout of vaccines in certain demographic areas, and the surge of variants with increased immune evasion, replicative fitness, and transmission [3,4]. Elucidating host-pathogen interactions that are critical for SARS-CoV-2 replication can facilitate the understanding of SARS-CoV-2 biology and the development of host-directed antivirals that could benefit from broad-spectrum activities and reduced viral resistance [5,6].

SARS-CoV-2 belongs to the family of enveloped viruses known as *Coronaviridae* [7], which are enveloped, positive-strand RNA viruses [8]. Virions are spherical and decorated with Spike (S) glycoproteins, which mediate receptor binding to facilitate viral entry [9]. Upon internalization, the viral RNA is released into the cytoplasm and transcribed into viral proteins [10]. These include structural proteins S, Envelope (E), Nucleocapsid (N), and Membrane (M) proteins, as well as 16 non-structural and 9 accessory proteins that are important for viral replication, innate immune evasion, and pathogenesis [11,12]. Coronaviruses induce the formation of double-membrane vesicles to promote the replication and transcription of their genomes [13]. Newly synthesized genomic RNAs are incorporated into virions and, following budding, infectious viruses are released from the host cell. Throughout their entire replication cycle, coronaviruses co-opt host factors that provide essential activities, including the cellular receptor ACE2 that is required for viral entry [14]. Several CRISPR functional genetic screens have illuminated host factors and cellular pathways that are required for replication of SARS-CoV-2 and other coronaviruses [15–26]. However, these CRISPR screens were conducted either in a pooled format, biasing them to the identification of host factors affecting initial stages of viral replication [15–25], or focused on the characterization of antiviral factors [26]. In addition, the majority of these screens used simian cells [15,22], or human cells ectopically expressing ACE2 [16,18,22,24]. Therefore, the host factor requirements for SARS-CoV-2 egress and budding in human cells that are naturally permissive for SARS-CoV-2 remain poorly characterized.

Here, we report findings of an arrayed genome-wide siRNA screen using human epithelial intestinal Caco-2 cells to identify host factors involved throughout the entire SARS-CoV-2 infectious cycle. These factors were subsequently validated using targeted CRISPR-Cas9 technologies and integrated with previously reported OMICs, including functional genetics and proteomics, to reveal pathways with support from multiple studies, including Wnt signaling or gap junction regulators, networks only identified in this study, including NADPH oxidation, or pathways supported by this study and previous proteomics studies but not functional screens, including arachinodate production and MAPK activity, which were mapped to assisting SARS-CoV-2 replication and egress. In addition, we identified 17 host factors required for the replication of SARS-CoV-1, -2, and MERS-CoV, including perlecan, which was found to facilitate viral entry and was determined as a direct interactor of SARS-CoV-2 S protein. Small molecules targeting the proviral factor Baculoviral IAP Repeat Containing 2 (BIRC2) were found to inhibit SARS-CoV-2 infection in a dose-dependent manner. The proviral effects of BIRC2 on SARS-CoV-2 growth were further confirmed in vivo by treating infected mice with a BIRC2 inhibitor. Overall, this study provides new insights into host factors required for the entire SARS-CoV-2 replication cycle, including late stages, and identifies SMAC mimetics as promising host-targeting inhibitors that can serve as the basis for new anti-SARS-CoV-2 therapies.

## Results

### Genome-wide screen identifies host factors involved in SARS-CoV-2 replication

**Fig 1:**
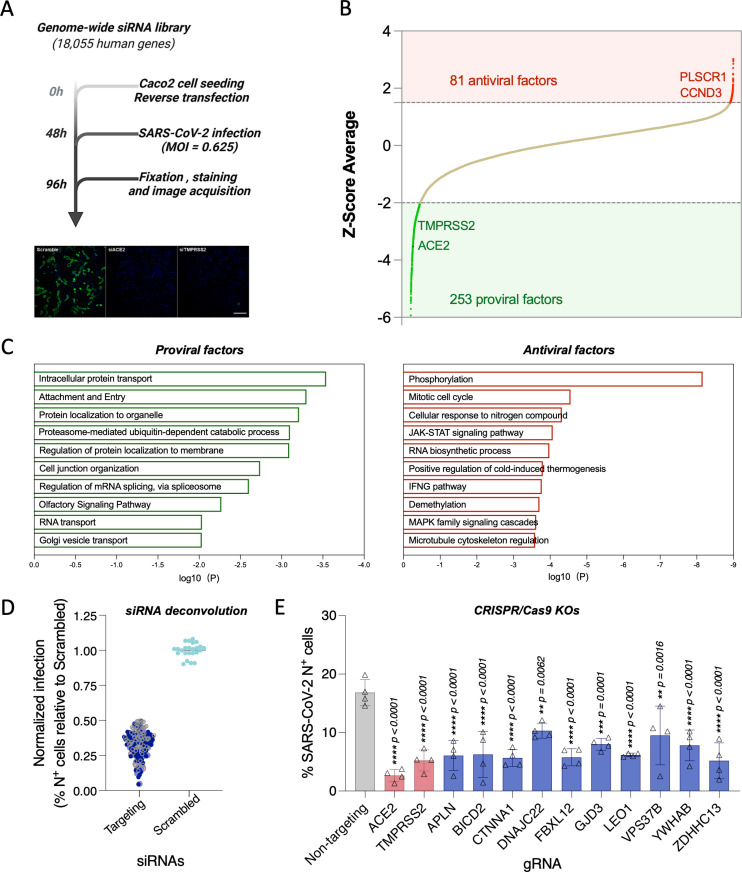
Genome-wide siRNA screen identifies host factors involved in SARS-CoV-2 replication. **(A)** Schematic representation of the genome-wide screen to identify human host factors that affect SARS-CoV-2 replication. **(B)** Ranked SARS-CoV-2 infectivity Z-scores from the genome-wide siRNA screen. Dashed lines illustrate cut-offs for hit calling strategy: Z-score ≤ −2 indicates proviral factors (green), Z-score ≥ 1.5 indicates antiviral factors (red). Controls are shown (e.g., siACE2, positive). **(C)** Functional enrichment analysis of identified proviral (*left-green*) and antiviral (*right-red*) host factors. **(D)** Deconvolution plot showing proviral host factors validated with one siRNA (gray), two siRNAs (dark blue), three siRNAs (light blue), and four siRNAs (pink). **(E)** Calu-3 cells treated with indicated gRNAs were infected with SARS-CoV-2 (MOI = 0.75) for 48 h prior to immunostaining for viral N protein. Shown is quantification of the normalized infection (% of SARS-CoV-2 N^+^ cells) relative to parental cells. Data show mean ± SD from one representative experiment in quadruplicate (*n* = 4) of two independent experiments. Significance was calculated using one-way ANOVA with Dunnett’s post-hoc test. The data underlying this figure can be found in S1 Data.

The systematic identification of cellular factors that either support or restrict viral replication can provide valuable insights into SARS-CoV-2 biology, pathogenesis, and identify new host-directed antiviral targets that might pose a higher barrier to resistant viruses. To uncover host factors involved in SARS-CoV-2 replication, we conducted a genome-wide siRNA screen in human Caco-2 cells challenged with USA-WA1/2020, the first SARS-CoV-2 US isolate (Fig 1A). This colorectal adenocarcinoma cell line was selected for the screen because the intestinal epithelium is a target for SARS-CoV-2 [27,28], and these cells endogenously express ACE2 and TMPRSS2, rendering them permissive to SARS-CoV-2 infection [14]. Furthermore, the siRNA knockdown efficiency is higher in Caco-2 cells compared to other SARS-CoV-2 permissive cell types such as Calu3. Cells were transfected with individually arrayed siRNAs, infected with SARS-CoV-2 for 48 h to allow for multicycle replication, immunostained for SARS-CoV-2 N protein, stained with DAPI, and then subjected to high content microscopy (Fig 1A). The impact of each individual gene knockdown on viral replication (% infected cells) was quantified based on DAPI^+^ events (number of cells) and SARS-CoV-2 N^+^ events (number of infected cells), and then normalized to the median % infection of each plate. Non-targeting, scramble siRNAs were included on each plate as negative controls, and siRNAs targeting SARS-CoV-2 entry factors ACE2 and TMPRSS2 were included as positive controls (S1A Fig). Screens were conducted in duplicate and showed good reproducibility with a Pearson correlation coefficient (*r*) = 0.66 (S1B Fig). Primary screening data were subjected to an analysis pipeline to identify siRNAs that affect viral replication (ranked based on Z-score) without impacting cell viability (cell count at least 70% of scramble control). Using these criteria, we identified 253 proviral host factors (including 222 with Z-scores < −2 in both replicates, and 31 with Z-score < −2 in replicate 1 and <−1.5 in replicate 2) (Fig 1B, green). Additionally, we identified 81 factors that restricted viral replication (Z-score > 1.5 in both replicates), including CCND3 and PLSCR1, which have been previously identified as restriction factors for SARS-CoV-2 [26,29] (Fig 1B, red). Findings are summarized in S1 Table. Reactome and gene ontology (GO) analyses of proviral factors revealed enrichment in intracellular protein transport (Log*P* = −3.5398), proteosome-mediated ubiquitin process (Log*P* = −3.1010), and Golgi vesicle transport (Log*P* = −2.0312), among the top 10 enriched terms (Fig 1C, left). Antiviral factors were enriched in protein phosphorylation (Log*P* = −8.1590), JAK-STAT signaling (Log*P* = −4.0693), and demethylation (Log*P* = −3.7072), among others (Fig 1C, right). Gene membership to these terms is included in S1 Table. To assess the overlap between these factors promoting SARS-CoV-2 infection and those identified in previous CRISPR screens, pairwise relative overlaps were calculated as the ratio of shared genes (intersection) to the total unique genes (union) between two datasets. We leveraged the comparative analysis performed by Grodzki and colleagues 2022 [22], who systematically reanalyzed previously published SARS-CoV-2 genome-wide CRISPR screens [16–18,20] and assessed the overlap with genes identified in our primary screen. For each dataset, the average relative overlap was calculated by averaging its pairwise overlaps with all other datasets. Z-scores were calculated to compare a dataset’s average relative overlap relative to the group mean, with positive scores indicating greater-than-average overlap and negative scores indicating less-than-average overlap, measured in units of standard deviation (S1C Fig). As expected, considering the differences in experimental conditions (arrayed versus pooled screening), cell types used, and libraries (siRNA versus CRISPR), the screen in this study exhibited the highest degree of unique factors (overlap = 0.0027, Z-score −1.06) compared with all other analyzed screens, suggesting for its potential to identify previously uncovered host factors for SARS-CoV-2 (S1C Fig). A list of all the unique host factors is included in S2 Table.

Next, host factors identified in the primary screen were subjected to a subsequent round of siRNA validation using four individually arrayed siRNAs per gene to minimize off-target effects. Here, 125 cellular factors were confirmed to affect the replication of SARS-CoV-2 with two or more siRNAs (Fig 1D and S3 Table) and their expression was verified across different relevant cell types [30], including primary mucocilliated epithelial cells, which are a known target of SARS-CoV-2 (S2 Fig). To further validate the impact of these factors on SARS-CoV-2 replication, we prioritized 10 factors based on potency and/or by integration with OMICs profiling of SARS-CoV-2 proteome and phosphoproteome [31–33], and validated their requirement for SARS-CoV-2 replication using CRISPR-Cas9 knockout in the human lung cell line Calu-3 (Fig 1E).Taken together, these data provide a list of validated host factors across different cell types that are involved in SARS-CoV-2 replication.

### Network integration reveals Wnt signaling, MAPK signaling, and NADPH oxidase regulation as relevant networks implicated in SARS-CoV-2 replication

**Fig 2:**
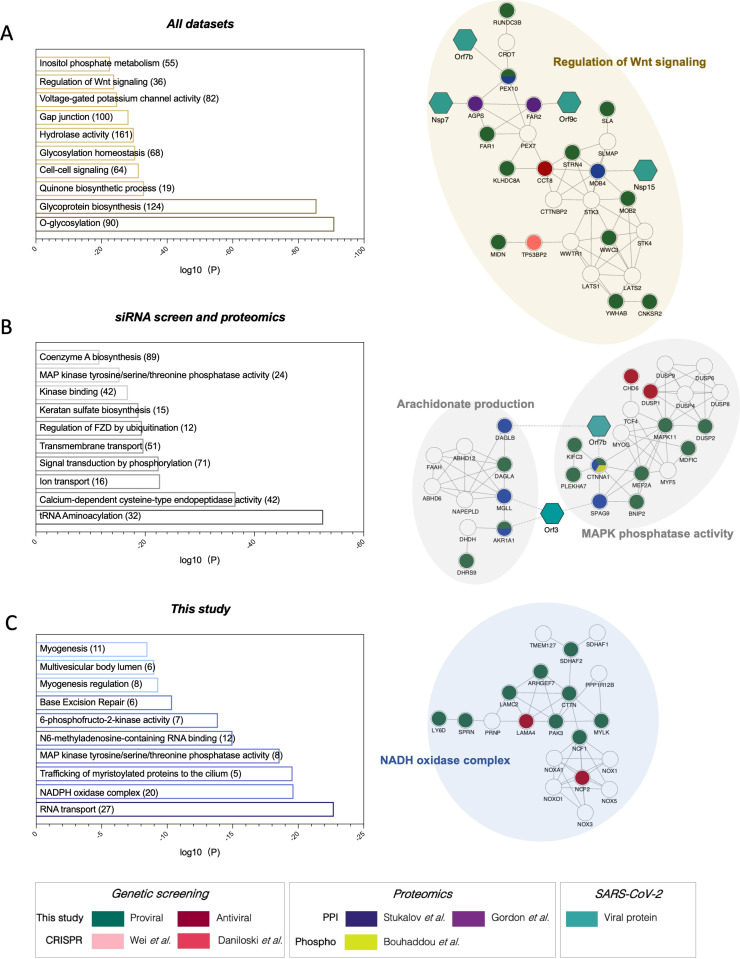
Network integration reveals relevant networks implicated in SARS-CoV-2 replication. The network containing the identified proviral (**green**) and antiviral (**red**) human host factors was integrated with host factors reported to be relevant for SARS-CoV-2 infection. These include genetic CRISPR screen hits (Wei and colleagues, 2020 [15], **light pink**; Daniloski and colleagues, 2020 [16], **dark pink**), protein–protein interaction hits (Stukalov and colleagues, 2020 [31], **blue**; Gordon and colleagues, 2020 [32], **purple**), as well as hits from a phosphoproteomics study (Bouhaddou and colleagues, 2020 [33], **yellow**). The network was subjected to supervised community detection [67,73], and then clustered based on identification by all datasets **(A)**, this screen and proteomics data but not CRISPR screens **(B)**, or exclusively by this screen **(C)**. The top 10 GO categories with the lowest *p*-values are shown. Continuous black edges indicate interactions from STRING database, discontinued edges indicate virus-host interactions. **Turquoise** nodes indicate SARS-CoV-2 proteins. **White** denotes proteins in network (based on STRING) but not identified in any of the OMICs studies. * indicates highlighted clusters.

SARS-CoV-2 relies on a number of cellular proteins to complete its replication cycle, from surface receptors for viral entry to vesicle transport and sorting proteins for viral trafficking and release [34]. Conversely, in response to infection, the cell activates an antiviral program to clear infection [29]. A network integration model was generated to identify the interactomes and networks that the SARS-CoV-2 proviral and antiviral factors identified in our primary screen belong to and thereby gain a better understanding of their role in viral replication. First, we conducted a supervised network propagation by creating a grid that included the siRNA screening hits and their high-confidence interactors as determined by the STRING database (see Methods). To place host factors that were identified in the context of previously identified SARS-CoV-2 host factors and highlight more confidence networks and host factors, we leveraged the first two reported SARS-CoV-2 functional genetic screens [15,16], as well as the first two reported SARS-CoV-2 interactome and a phosphoproteomics datasets [31–33]. These datasets were integrated with the genetic screen data generated in this study, and community detection algorithms were applied to identify densely interconnected clusters of factors that show significant membership in biological processes (S3 Fig; see Methods). To highlight pathways with support from all these studies, and thus likely relevant during SARS-CoV-2 infection as well as unique for this study, the resulting hierarchical ontology network was divided into three clusters. The first cluster included enrichment in pathways supported by factors found in all datasets. Here, the top 10 pathways revealed enrichment in gap junction (log10(*P*) = −28.2433), as well as Wnt signaling (log10(*P*) = −23.8542) (Fig 2A—left). Within this pathway, the proviral peroxidase factor PEX10 was found to interact with SARS-CoV-2 Orf7b and be in complex with perixomal regulator FAR1 and Nsp7 interactor AGPS (Fig 2A—right). The second cluster encompassed factors found in our siRNA screen and the proteomics datasets, but not previously published CRISPR functional screens (Fig 2B—left). This cluster included enrichment in pathways like MAPK phosphatase activity (log10(*P*) = −15.3043), where the cell adhesion molecule *CTNNA1* was found to interact with SARS-CoV-2 Orf7b protein and to be phosphorylated in response to infection, or arachidonate production (log10(*P*) = −16.7445), where the alcohol dehydrogenase AKR1A1 was found to support SARS-CoV-2 replication and to interact with Orf3 (Fig 2B—right). Finally, the last cluster included pathways supported exclusively by this screen (Fig 2C). Here, we found enrichment in RNA binding molecules, including base excision repair (log10(*P*) = −10.3733), or NADPH oxidase regulation (log10(*P*) = −19.6374) (Fig 2C—left), which included several Laminin proteins as well as neutrophil cytosolic factors both as positive and negative regulators of SARS-CoV-2 infection (Fig 2C—right). To enable further analyzing these data, we uploaded all these networks and parental interactomes into the web interface NDEx (see Data availability statement). Overall, these analyses revealed host factors and networks that are supported by one or more OMICs datasets, thus providing a higher level of confidence and more insight into their mechanism of proviral or antiviral action.

### Mapping of host factors into SARS-CoV-2 infectious cycle reveals a direct interaction between perlecan and SARS-CoV-2 S protein

**Fig 3:**
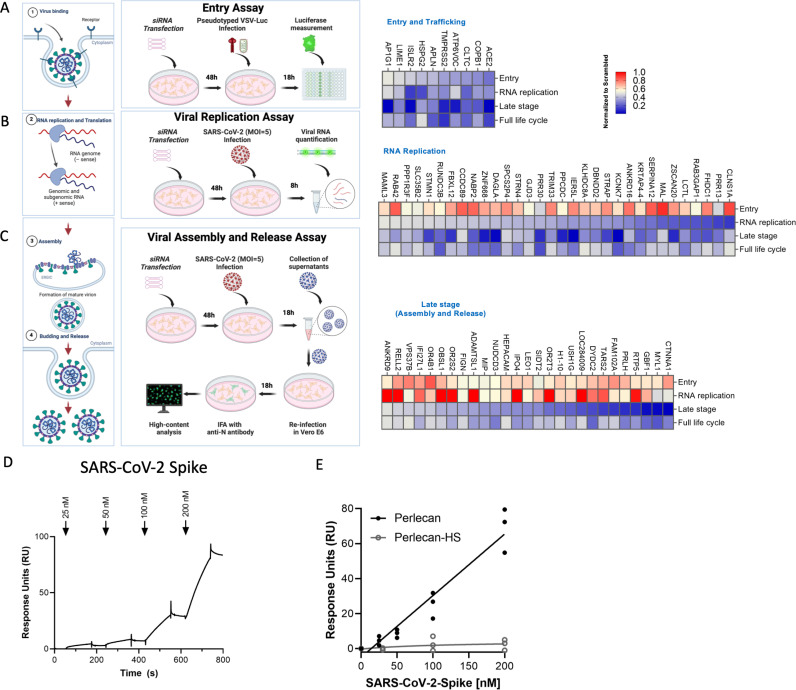
Mapping of host factors into the SARS-CoV-2 replication cycle reveals a direct interaction between entry factor perlecan and SARS-CoV-2 S protein. **(A)** Caco-2 cells were subjected to siRNA-mediated knockdown of indicated host factors and then infected with SARS-CoV-2 pseudotyped VSV luciferase virus (VSV-S-luc) for 18 h prior to measurement of luciferase signal. **(B)** In parallel, cells were subjected to synchronized infection with SARS-CoV-2 (MOI = 5) for 8 h prior to measurement of viral RNA, or **(C)** supernatants collected at 18 h post-infection were used to infect naïve Vero E6 cells. The % of infected cells was then determined at 18 h post-infection using immunostaining for viral N protein (3−4). In parallel to these experiments, the impact of depleting these factors on SARS-CoV-2 replication was evaluated at 24 h post-infection in Caco-2 cells (full replication cycle, Fig 3A–3C). Results are summarized in the heat map and show the mean (*n* = 2) of relative activities compared to cells treated with non-targeting scramble siRNA. **(D, E)** Surface plasmon resonance (SPR) was used to evaluate the binding of S protein to perlecan or perlecan without HS binding to immunopurified perlecan isolated from human coronary artery endothelial cells. Control flow channels contained immobilized BSA. S protein at indicated concentrations was run across the flow channels for 120 s and dissociation was measured in the following 600 s. The RU values throughout the experiment for BSA were subtracted from the RU values for perlecan to determine the level of specific binding. This experiment was repeated with perlecan treated with heparinase III. The data underlying this figure can be found in S1 Data.

The proviral host factors that were found to affect replication of SARS-CoV-2 with two or more siRNAs were evaluated for their effect during the three main stages of the SARS-CoV-2 infectious cycle: entry, replication, and assembly/egress. First, to identify host factors involved in viral entry, siRNA-transfected Caco-2 cells were infected with a vesicular stomatitis virus (VSV) encoding luciferase, pseudotyped with either SARS-CoV-2 S protein or VSV Glycoprotein (G), and luciferase levels were measured as indicators of entry. siRNA-mediated knockdown of *ACE2*, *TMPRSS2*, *COPB1*, *ATP6V0C*, *CLTC*, *APLN*, *HSPG2*, *IRLR2*, *LIME1*, and *AP1G1* significantly reduced entry mediated by SARS-CoV-2 S protein (Fig 3A). Of these, *CLTC* and *COPB1* were also found to participate in VSV-G mediated entry (S4A Fig), suggesting that both SARS-CoV-2 and VSV hijacked clathrin-mediated endocytosis to enter the host cells. Notably, the other eight factors showed no effect on VSV-G-mediated entry (Figs 3A and S4A), including *TMPRSS2* or transmembrane protein *LIME1*, suggesting they are specific for SARS-CoV-2 S-dependent entry.

*HSPG2*, also known as Perlecan, was found to be important for SARS-CoV-2 entry (Fig 3A). Perlecan is a large multi-domain extracellular matrix proteoglycan, commonly found in all native basement membranes [35]. Interestingly, Heparan sulfate (HS) has been shown to act as a co-receptor or an attachment factor for a number of viruses, including SARS-CoV-2 [36,37], and is a common modification found on Perlecan. Given the dependence of SARS-CoV-2 on both HS and Perlecan for viral entry, we investigated role of heparin sulfanation of Perlecan for viral attachment. Therefore, we isolated Perlecan from human coronary artery endothelial cells as previously described [38] and measured its interaction with recombinant full-length S protein and its receptor binding domain (RBD) using a biacore biosensor. Both S and S RBD bound to Perlecan but not albumin (negative control) (Figs 3D and S4B–S4D), although the interaction was more significant with full-length S (Figs 3D and S4D). Treatment of the isolated Perlecan with an HSase eliminated binding, showing that the S protein interacts with the HS chain and not the core protein (Fig 3E). This is in agreement with previous data showing that HS is required for S binding to cells [37]. Collectively, this data support a critical role for direct heparin sulfate modification of HSPG2 for SARS-CoV-2 attachment and entry.

SARS-CoV-2 replication involves the early translation and polyprotein processing of incoming genomic RNAs to enable viral RNA synthesis [39]. Thus, to define host factors that affect SARS-CoV-2 RNA replication and translation, Caco-2 cells knockdown for each target gene were infected with SARS-CoV-2 at MOI = 5 and viral RNA levels were then quantified at 8 h post-infection (Fig 3B). Relative RNA replication was calculated by comparing the viral RNA copy number in targeted siRNA-transfected cells by that in scramble cells. This assay revealed 32 host factors that strongly inhibit SARS-CoV-2 RNA replication (>50% inhibition) but do not affect viral entry. These include RNA-binding protein STRAP, which was previously reported as a SARS-CoV-2 interactor [31], and the ubiquitin ligase FBXL12, a reported interactor of SARS-CoV-2 Orf8 [32]. Interestingly, within these 32 factors, we found several mapped to pathways not identified by previous CRISPR screens, including coenzyme A biosynthesis regulators ZNF688, PPCDC, and ZSCAN20 (Fig 2B, left), arachidonate regulator DAGLA, which was found to be in network with SARS-CoV-2 Orf3, and kinase binding regulator MAL (Fig 2B, left).

Lastly, to identify factors involved in the late stages of the viral cycle, we infected naïve Vero-E6 cells with viral supernatants that were collected at 18 h post-infection of siRNA-transfected Caco-2 cells (Fig 3C) followed by immunostaining for viral N protein. We found that depletion of 27 host factors lowered by >50% the amount of infectious viral particle production without affecting viral entry or RNA replication, suggesting that they specifically participate in the late stages of SARS-CoV-2. These include the lysosomal protein SIDT2, which is in agreement with previous reports showing that SARS-CoV-2 hijacks lysosomes for egress [40], the member of the PAF complex LEO1, shown previously to be targeted by influenza A virus to suppress the antiviral response [41], and the Golgi resident and vesicle trafficking protein GBF1, a previously reported interactor of SARS-CoV-2 M [31] (Fig 3C). Interestingly, we found enrichment in pathways not found by previous CRISPR screens, including MAPK phosphatase activity, where the adhesion molecule CTNNA1 was found to be phosphorylated during infection and to interact with Orf7b, as well as factors not previously reported, including the calcium-dependent endopeptidases OBSL1 and MYL1, found in network with SARS-CoV-2 M protein (Fig 2B).

### Comparative screening reveals proviral factors conserved across several coronaviruses

**Fig 4:**
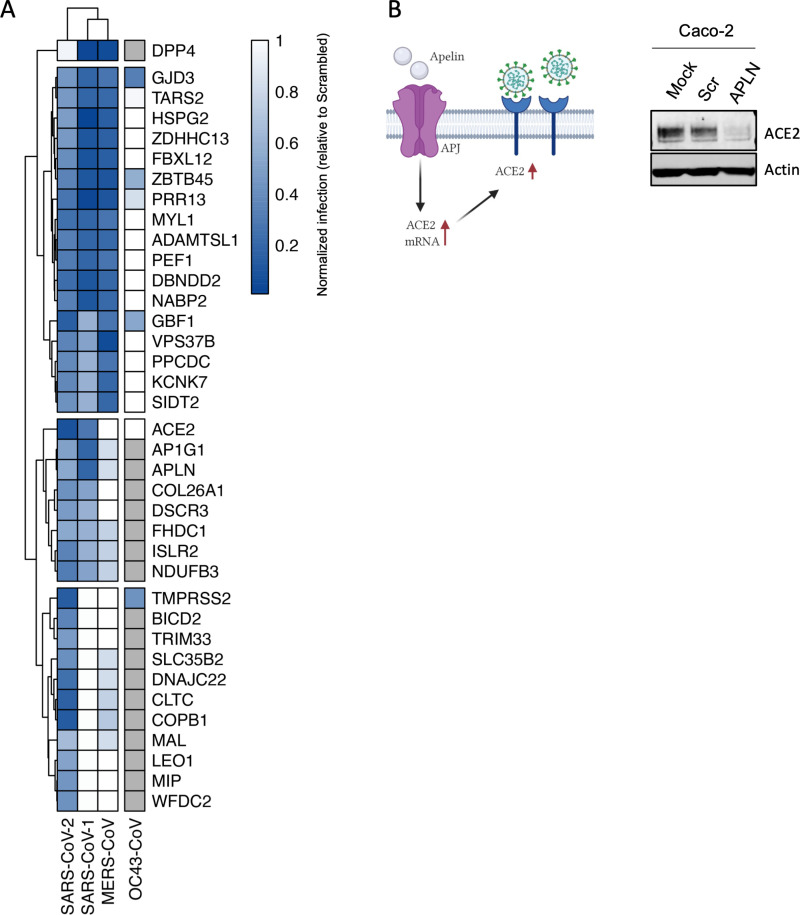
Comparative screening reveals proviral factors that are conserved across several Coronaviruses. **(A)** Heat map showing normalized infection of SARS-CoV-1, CoV-2, MERS-CoV, and OC43-CoV upon knockdown of indicated human host factors. Caco-2 cells depleted for indicated factors were infected with SARS-CoV-2 (MOI = 0.625) for 48 h prior to immunostaining for viral N protein. Shown is quantification of the normalized infection (% of SARS-CoV-2 N^+^ cells) relative to control cells (scrambled siRNA). A549-DPP4, A549-ACE2, or A549 were depleted for indicated factors and then infected with MERS-CoV, SARS-CoV-1, or OC43-CoV respectively (MOI 0.1). At 48 h post-infection, supernatants were collected and used to calculate the TCID50. Data shows TCID50/ml relative to control cells (scrambled siRNA). Data show mean ± SD from one representative experiment in duplicate (*n* = 2) of two independent experiments. Cells in gray mean the factor was not tested. **(B)** Cell lysates from Caco-2 cells mock-treated or treated with scrambled or APLN siRNAs for 48 h were then subjected to SDS-PAGE and immunoblotted using antibodies specific for ACE2 and Actin (loading control). Blot is representative of two independent experiments. The data underlying this figure can be found in S1 Data. The original uncropped blots can be found in S1 Raw Images.

Motivated by the premise that the identification of host factors essential for replication of several related viruses might inform broad-acting antiviral therapies, we prioritized 47 validated SARS-CoV-2 proviral host factors based on their level of activity, multiOMIC support, and mapping data, and evaluated their impact on SARS-CoV-1 and MERS replication. From these, 17 factors were required for all three coronaviruses, suggesting these factors might be required for beta-coronavirus replication (Fig 4A). These include the palmitoyltransferase ZDHHC13, which has been linked to S-mediated syncytia formation and viral entry [42], the mitochondrial TARS2, a reported interactor of SARS-CoV-2 M protein [32], and the sorting protein VPS37B, which was previously associated with HIV-1 budding [43], and was found in our analysis to affect SARS-CoV-2 egress (Fig 3C). From these 17 factors, 3 factors (the transcription regulator ZBTB45, and the homeostasis regulators GJD3, and GBF1) were also found to support the common coronavirus OC43, suggesting their potential to be pan-coronavirus targets (Fig 4A). In addition, eight host factors, including ACE2, AP1G1, and ACE2 positive regulator APLN, whose knockdown reduced ACE2 protein levels [44] (Fig 4B), were required for SARS-CoV-1 and SARS-CoV-2 infection, but had limited effects on MERS-CoV infection, and 11 factors were found to be exclusively required for SARS-CoV-2 replication. However, since these replication assays were carried out across different cellular backgrounds, additional studies will be required to understand the specificity of these factors. Collectively, these data have revealed a subset of host factors that are conserved across these three coronaviruses and have the potential to lay the groundwork for broad-acting anti-coronavirus therapies.

### Pharmacological inhibition of BIRC2 reduces SARS-CoV-2 replication in vitro and in vivo

**Fig 5:**
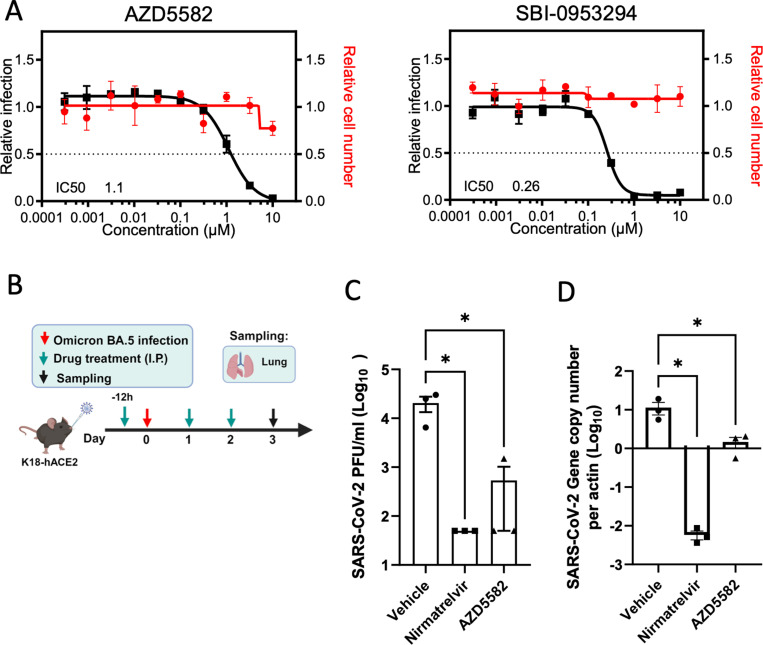
Pharmacological inhibition of BIRC2 reduces SARS-CoV-2 replication in vitro and in vivo. **(A)** Dose–response analysis of SBI-0953294 and AZD5582 showing infectivity (black), cell number (red), and cellular IC_50_ values. **(B)** Layout of mice experiments. Effect of AZD5582 on SARS-CoV-2 Omicron replication in the lungs of infected mice as measured by plaque assay **(C)** and qRT-PCR **(D)**. Tissue sampling was done at 72 hpi. One-way ANOVA when compared with the vehicle control group, *p < 0.05. And the detection limit = 50 PFU/ml in a 12-well plate. The data underlying this figure can be found in S1 Data.

Host-directed antivirals represent a promising approach for the development of escape-resistant therapies. BIRC2 was one of the proviral host factors identified in our screen (S1 Table). We previously reported BIRC2 as a critical host factor involved in HIV-1 transcription through its role as a repressor of the non-canonical NF-κB pathway, and we and others have developed bioavailable small molecules to target this protein [45–47]. Degradation of BIRC2 results in the accumulation of NF-κB-inducing kinase and the proteolytic cleavage of p100 into p52, so that p52 can then bind the RELB transcription factor to undergo nuclear translocation and induce the expression of target genes [48]. To evaluate whether pharmacological inhibition of BIRC2 had an impact on SARS-CoV-2 replication, we employed two different BIRC2-specific small molecule antagonists, known as Smac mimetics, AZD5582 and SBI-095329 [45,49]. First, we validated the impact of BIRC2 inhibition on NF-κB signaling as treatment of Caco-2 cells with AZD5582 resulted in cleavage of p100 to p52 in a dose-dependent manner (S5A Fig). Importantly, we also confirmed that treatment with either AZD5582 or SBI-095329 reduced SARS-CoV-2 infection in a dose-dependent manner without inducing cytotoxicity (Fig 5A). To further evaluate the impact of BIRC2 inhibition on SARS-CoV-2 replication in vivo, mice were pre-treated with the commercially available AZD5582 (3 mg/kg), Nirmatrelvir (200 mg/kg), or DMSO (control) and then infected with SARS-CoV-2 (Omicron BA.5 and Alpha B.1.1.7) (Figs 5B and S5B). Although prolonged treatment (6 days) with AZD5582 was not well tolerated and resulted in a significant reduction in mice body weight and survival (S5C and S5D Fig), at 3 days post-infection treatment with AZD5582 significantly reduced SARS-CoV-2 viral titers and RNA copy number in the lung both for Omicron and Alpha variants (Figs 5C, 5D, and S5E). Combined, these data show that BIRC2 positively impacts SARS-CoV-2 replication in vitro and in vivo, suggesting its potential as a druggable target for SARS-CoV-2 treatment.

## Discussion

In this study, we carried out a genome-wide siRNA screen to identify host factors involved throughout the complete SARS-CoV-2 infectious cycle, from attachment and entry to release of viral particles. These data were able to highlight host factors, and networks revealed by multiple previously published OMICs studies that are required for the replication of SARS-CoV-2 and other coronaviruses, thus constituting relevant therapeutic targets for host-directed antivirals. Additionally, the unique configuration of the arrayed RNAi screen enabled us to highlight factors, networks, and pathways identified exclusively by this study.

Since the beginning of the COVID-19 pandemic, 10 whole-genome pooled CRISPR screens and one arrayed CRISPR screens have been conducted to identify host factors involved in SARS-CoV-2 replication. Overall, the majority of these screens used different cell lines of simian (Vero E6 [15,25]) or human origin, many of which were engineered to ectopically express ACE2 (A549, Huh7.5, HEK-293 [16–18,22,24]) to support SARS-CoV-2 growth. Here, we utilized colorectal epithelial Caco-2 cells, as the intestine is a target of SARS-CoV-2, and these cells are naturally permissive for SARS-CoV-2 [50]. Advantages of arrayed siRNA screening include sensitivity, as we measured viral growth at a single cell resolution in over 3,000 cells per siRNA rather than sequencing surviving cells, which was the approach of many screens, as well as the ability of knockdown screening to mimic more closely the conditions achieved by drug treatment. In addition, pooled CRISPR screens tend to be biased towards identifying factors that play a role in the early stages of the viral cycle, but arrayed siRNA screens do not show this bias and capture the entire replication cycle. Accordingly, we found that 40% (4 out of 10) of the host factors identified in this study could be assigned to the early steps of the cycle were described in at least one pooled CRISPR screen, while only 6% (2 out of 32) and 4% (1 out of 27) of the hits mapped to replication or the late stages, respectively, were identified as top hits in those screens (S4 Table). Considering that 85.5% of the host factors identified by the siRNA screen were found to affect post-viral entry stages (Fig 3), including coenzyme A biosynthesis, arachidonate production, kinase binding, or MAPK signaling, pathways which were not previously identified by previous CRISPR screens, these data provide novel insights into the poorly understood host factors required for SARS-CoV-2 assembly, trafficking, and budding.

Integration of OMICs datasets can reveal host factors and networks with multiOMIC support thereby increasing the likelihood that they are critical for SARS-CoV-2 replication. In particular, integration of the data generated in this study with published CRISPR functional screens and proteomics—including protein–protein interactions (PPI) and phosphoproteomics—revealed enrichment in regulation of Wnt signaling, gap junction, glycosylation homeostasis, cell–cell signaling, and epigenetic regulation. (Figs 2A and S3). In fact, several groups have reported critical physical and functional interactions between SARS-CoV-2 and other viruses with the Wnt signaling machinery to promote viral survival [51], the role of glycosylation to enable S-mediated entry and stimulate innate immune activation [52], or the ability of SARS-CoV-2 to hijack MAPK11 to promote viral replication [53]. Less understood is the role of epigenetic regulation during SARS-CoV-2. Although it may seem surprising that a cytoplasmic virus relies on nuclear factors to complete its infectious cycle, several cytoplasmic RNA viral proteins undergo nuclear translocation, can mislocalize nuclear proteins into the cytoplasm, or rely on the cytoplasmic products of nuclear transcription factors or associated proteins [54–56]. In addition, recent work showed that SARS-CoV-2 variants of concern have gained the ability to interact with members of the gene transcription regulator PAF complex [57], including LEO1, which was found as a validated host factor in our screen (Fig 1E). However, more work will be required to understand the functional consequences of these interactions and mechanism of action.

Among the factors found to affect SARS-CoV-2 entry was HSPG2 (Perlecan, Fig 3A). Perlecan is a large, multi-domain proteoglycan modified by HS that is located in the extracellular matrix (ECM) and basement membranes of the airway and alveolar epithelia and could therefore directly abet SARS-CoV-2 infection [35]. Studies utilizing enzymatic degradation of HS or using competitive inhibitors that block the binding sites of HS have demonstrated reduced infection rates of SARS-CoV-2 in cell cultures [58]. Furthermore, variations in the structure of HS chains can affect the efficiency of viral attachment and entry, indicating a level of specificity in the interaction between HS and SARS-CoV-2. The involvement of HS in the entry mechanism of SARS-CoV-2 is also consistent with their known roles in the entry of other viruses [59]. We employed Surface Plasmon Resonance (SPR) and revealed Perlecan as a direct interactor of SARS-CoV-2 S protein in an HS-dependent manner, thus adding to the growing evidence that specific HS-modified proteins could participate in SARS-CoV-2 entry. Further understanding of this mechanism could lead to broad-spectrum antiviral strategies targeting the initial attachment phase of viral infection.

Another potential mechanism of broad-acting viral inhibition is targeting the inhibitor of apoptosis proteins (IAPs), which play key and complex roles in innate immunity, inflammation as well as the regulation of cell death and cell proliferation [60,61]. Smac mimetics inhibit IAPs and have been recognized as potent HIV-1 latency reversal agents [45], and more recently described to have antiviral properties [49]. In this study, we found two Smac mimetics, AZD5582 and SBI-095329, that through inhibition of the proviral host factor BIRC2, conferred antiviral properties in vitro against the ancestral Wuhan-1 SARS-CoV-2, and in vivo (AZD5582) across the two variants of concern Omicron and Alpha. Although no toxicity was recorded in our in vitro experiments, prolonged treatment in mice resulted in reduced survival and body weight, suggesting more work will be required to address their safety profile. Importantly, a recent publication showed that the Smac mimetic BI–82 conferred antiviral activities across dengue, zika, and hepatitis B virus in vitro, and was well-tolerated and showed potent efficacy against influenza A virus in vivo [49]. Combined with our data, this suggests that the expression program governed by non-canonical NF-κB signaling potently restricts SARS-Cov-2 replication both in vitro and in vivo, and further underscores the potential of Smac mimetics as broad-acting antiviral therapies.

In summary, our study unveils novel host factors that are critical for all three main stages of SARS-CoV-2 infectious cycle. Importantly, we carried out comparative screening across SARS-CoV-1, MERS, and OC43-CoV highlighting commonalities that could inform the development of host-directed, pan-coronaviral antiviral therapies. Further characterization of host proteins elucidated through this analysis will provide additional molecular insights into the host machinery that governs SARS-CoV-2 replication and pathogenesis, and provides a resource for the evaluation of additional proteins as potential host-directed therapeutic targets using additional ex vivo and in vivo models, as well as their evaluation as potential determinants of disease susceptibility.

## Methods

### Ethics statement

All experimental protocols were approved by the Animal Ethics Committee in the University of Hong Kong (CULATR) and were performed according to the standard operating procedures of the biosafety level 3 animal facilities (Reference code: CULATR 5754-21).

#### Cells and viruses.

SARS-CoV-2 USA-WA1/2020, isolated from an oropharyngeal swab from a patient with a respiratory illness who developed clinical disease (COVID-19) in January 2020 in Washington, USA, was obtained from BEI Resources (NR-52281). These viruses were propagated using Vero E6 cells, collected, aliquoted, and stored at −80 °C. Plaque forming unit (PFU) assays were performed to titrate the cultured virus. All experiments involving live SARS-CoV-2 followed the approved standard operating procedures of the Biosafety Level 3 facility at the Sanford Burnham Prebys Medical Discovery Institute. SARS-CoV-1 (MA15) was generated produced as decribed [62]. The Jordan MERS-CoV strain (GenBank accession no. KC776174.1, MERS-CoV-Hu/Jordan-N3/2012) was kindly provided by Kanta Subbarao (National Institutes of Health, Bethesda, MD) and Gabriel Defang (Naval Medical Research Unit-3, Cairo, Egypt). The OC43 CoV was obtained from the ATCC. All work with SARS-CoV-1, MERS, and OC43-CoV was performed in a Biosafety Level 3 laboratory and approved by the University of Maryland Institutional Biosafety Committee. Caco-2 (ATCC HTB-37), Vero E6 (ATCC CRL-1586), HEK293T (ATCC CRL-3216), Calu-3 (ATCC HTB-55), A549-DPP4 (kind gift from Susan Weiss, UPenn), and A549-ACE2 (kind gift from Brad Rosenburg, Mount Sinai) cells were maintained in cell growth media: Dulbecco’s modified eagle medium (DMEM, Gibco) supplemented with 10% heat-inactivated fetal bovine serum (FBS, Gibco), 50 U/mL penicillin—50 µg/mL streptomycin (Fisher Scientific), 1 mM sodium pyruvate (Gibco), 10 mM 4-(2-hydroxyethyl)-1-piperazineethanesulfonic acid (HEPES, Gibco), and 1× MEM non-essential amino acids solution (Gibco). All cells were regularly tested and were confirmed to be free of mycoplasma contamination.

### siRNA screening

A whole-genome wide ON-TARGETplus SMARTpool siRNA library (Dharmacon, each containing 4 siRNAs targeting an individual gene) was seeded at 0.5 pmol each/well in 384-well plates (Greiner). For reverse transfection, Lipofectamine RNAiMAX was added in 10 µL OPTI-MEM to each well at a final dilution of 1:100 using a Combi reagent dispenser, followed by addition of 3,000 Caco-2 cells in 40 µL complete media per well. 48h post-transfection, cells were challenged by SARS-CoV-2 at MOI 0.625. Forty-eight-hour post-infection, plates were fixed by 4% PFA in PBS for 4 h at room temperature, then permeabilized by 0.4% Triton X-100 in PBS for 15 min at room temperature. Plates were blocked by 10% goat serum in 3% BSA in PBS for 30 min at room temperature, followed by incubation of primary antibody against SARS-CoV-2 NP at 1,000 in 3% BSA in PBS at 4 °C overnight. Primary antibody inoculum was removed and plates were washed three times with PBS by plate washer, then incubated with anti-rabbit Alexa Fluor 488 (Invitrogen) at 1,000 in PBS for 1h at room temperature. Secondary antibody inoculum was removed and plates were washed three times with PBS by plate washer, then DAPI was added in PBS. Plates were then sealed and imaged using the Celigo Image Cytometer (Nexcelom).

### Generation of Calu-3 CRISPR/Cas9 knockouts

Detailed protocols for RNP production have been previously published [63]. Briefly, lyophilized guide RNA (gRNA) and tracrRNA (Dharmacon) were suspended at a concentration of 160 µM in 10 mM Tris-HCL, 150 mM KCl, pH 7.4. Five µL of 160 µM gRNA was mixed with 5 µL of 160 µM tracrRNA and incubated for 30 min at 37 °C. The gRNA:tracrRNA complexes were then mixed gently with 10 µL of 40 µM Cas9 (UC-Berkeley Macrolab) to form CRISPR-Cas9 ribonucleoproteins (crRNPs). Five 3.5 µL aliquots were frozen in Lo-Bind 96-well V-bottom plates (E&K Scientific) at −80 °C until use. Each gene was targeted by 4 pooled gRNA derived from the Dharmacon pre-designed Edit-R library for gene knock-out (sequences and catalog numbers provided in Table 1 below). Non-targeting negative control gRNA (Dharmacon, U-007501) was delivered in parallel. Each electroporation reaction consisted of 2.0 × 10^5^ Calu-3 cells, 3.5 µL crRNPs, and 20 µL electroporation buffer. Calu-3 cells were grown in fully supplemented MEM (10% FBS, 1×Pen/Strep, 1× non-essential amino acids) to 70% confluency, suspended and counted. crRNPs were thawed and allowed to come to room temperature. Immediately prior to electroporation, cells were centrifuged at 400*g* for 3 min, supernatant was removed by aspiration, and the pellet was resuspended in 20 µL of room-temperature SE electroporation buffer plus supplement (Lonza) per reaction. Twenty µL of cell suspension was then gently mixed with each crRNP and aliquoted into a 96-well electroporation cuvette for nucleofection with the 4-D Nucleofector X-Unit (Lonza) using pulse code EO-120. Immediately after electroporation, 80 µL of pre-warmed media was added to each well and cells were allowed to rest for 30 min in a 37 °C cell culture incubator. Cells were subsequently moved to 12-well flat-bottomed culture plates pre-filled with 500 µL pre-warmed media. Cells were cultured at 37 °C/5% CO_2_ in a dark, humidified cell culture incubator for 4 days to allow for gene knock-out and protein clearance prior to downstream applications.

**Table 1:** gRNA sequences used in this study.

Gene Symbol	Gene ID	gRNA Sequence	Catalog Number
Non-targeting	n/a	n/a	U-007501
ACE2	59272	GATGCAATGGTGGACCAGGT	CM-005755-01
ACE2	59272	GCATCCAATTGGACTGATAT	CM-005755-02
ACE2	59272	GCTTATTACTTGAACCAGGT	CM-005755-04
ACE2	59272	TACCAAGCAAATGAGCAGGG	CM-005755-03
TMPRSS2	7113	CAATGCCATGGATTGTTAAG	CM-006048-01
TMPRSS2	7113	CTATCCCGCACAGCCCACTG	CM-006048-03
TMPRSS2	7113	TTCCAGTCGTCTTGGCACAC	CM-006048-04
TMPRSS2	7113	AGCCGCCAGAGCAGGATTGT	CM-006048-02
APLN	8862	TACCTGCTTCAGAAAGGCAT	CM-017023-01
APLN	8862	AGAAAGGCATGGGTCCCTTA	CM-017023-02
APLN	8862	GAAAGGCATGGGTCCCTTAT	CM-017023-03
APLN	8862	TCTTCCAGCCCATTCCCATC	CM-017023-04
BICD2	23299	GTGGCTCAGACTTCAGGCTA	CM-014060-02
BICD2	23299	TGTCTGGCCAGCAGAATACA	CM-014060-01
BICD2	23299	GTGCTCAAAGCCATTGACCA	CM-014060-04
BICD2	23299	GAGGCCCTCAAACTCCACCT	CM-014060-03
CTNNA1	1495	GTGTCCAAATGGGACGACAG	CM-010505-02
CTNNA1	1495	GATGCCATCATATACCAGGC	CM-010505-03
CTNNA1	1495	GGATGCTGAAGTGTCCAAAT	CM-010505-04
CTNNA1	1495	GAGGGCGATGCGTTGCAGGT	CM-010505-01
DNAJC22	79962	ATGCTGGCGGCCACGCTAAT	CM-014507-01
DNAJC22	79962	TTTGCTGCCCAGGTGATAGT	CM-014507-02
DNAJC22	79962	AGTAGCCTCCAGATCCGGTA	CM-014507-03
DNAJC22	79962	GGCCACGCTAATGGGCAGTA	CM-014507-04
FBXL12	54850	GTGGCGGCTGATGGCCAGCA	CM-005204-02
FBXL12	54850	ATGCCATGTACCTTCGAAGG	CM-005204-04
FBXL12	54850	GATGGGCACCATGCTCAGGT	CM-005204-01
FBXL12	54850	ATGCGGATCCGGTCCCGTAC	CM-005204-03
GJD3	125111	GAGTAGACGACGAACAGCAC	CM-016720-01
GJD3	125111	GAAGAGCCAGAAGCGGTAGT	CM-016720-02
GJD3	125111	CTCTTGCTCGTCCTCGAACA	CM-016720-03
GJD3	125111	CTGCTCAGCGTAGCCGAGCT	CM-016720-04
LEO1	123169	AGACAAGGTACTGGTCTACA	CM-016579-01
LEO1	123169	CTGTGCTGATCTACATCTGA	CM-016579-02
LEO1	123169	CCTAATGATGATGAAGACGA	CM-016579-04
LEO1	123169	CCAAACAGTTCCTTATTACT	CM-016579-03
VPS37B	79720	AAGTGCTAACAGGGTCTCCA	CM-014404-04
VPS37B	79720	CTGCCTGAAGAAGTGCTAAC	CM-014404-02
VPS37B	79720	ACGCTTGACCCAGAAATACC	CM-014404-03
VPS37B	79720	CTGTAATCCTGGGTACGGCA	CM-014404-01
YWHAB	7529	GTGCCAGACCAAGACGAATT	CM-008766-01
YWHAB	7529	TGATATGGCTGCAGCCATGA	CM-008766-02
YWHAB	7529	GGCGCCTACCACATTCTTGT	CM-008766-03
YWHAB	7529	GTTGCCTACAAGAATGTGGT	CM-008766-04
ZDHHC13	54503	GTATGTGGCTGGATTATATA	CM-020510-02
ZDHHC13	54503	TATGTATCCAATAGCCCACA	CM-020510-04
ZDHHC13	54503	AACTGATCCAGGCTTCACTA	CM-020510-03
ZDHHC13	54503	CCACACAGCAGTTGCATACA	CM-020510-01

#### Network analyses.

*Rationale:* To understand the biochemical and functional context in which the identified host factors for SARS-CoV-2 function, we built a model that places these hits in known interactomes. A hierarchy of the clusters is generated wherein larger clusters are composed of smaller ones [64,65]. Unlike the human-curated GO, the structure is derived by the use of a multi-scale clustering algorithm applied to a reference PPI network, in this case, a high-confidence subset of the STRING database. To focus the model on the experimental data, it is built using the functional hits found in this study and their close neighbors. The interpretation of the experiment is performed by projecting the hits onto the clusters in the model, analogous to mapping them to GO terms [66]. Candidate names are proposed for each cluster by performing functional enrichment, finding the closest matching pathways and GO terms. Comparing this model to the result of a GO analysis, it has the advantages that its terms (clusters) are algorithmically derived from protein interactions that are in a sense “proximal” to the hits so that the hits can be investigated in the context of their underlying interactions.

*Approach:* To explore the highest confidence interactions of “hit” proteins, we selected the STRING - Human Protein Links - High Confidence (Score ≥ 0.7) PPI network available on NDEx as the “background” network (link provided below). We then performed network propagation to select a neighborhood of 300 proteins ranked highest by the algorithm with respect to these seeds [67]. This “neighborhood” network was extracted from the background network. We then identified densely interconnected regions, i.e., “communities” within the neighborhood network, using the community detection algorithm HiDeF via the Community Detection Application and Service (CDAPS) [68,69] (app available at [24^,^ 25]]. The result of HiDeF from CDAPS was a “hierarchy” network where each node represented a community of proteins, and edges denoted containment of one community (the “child”) by another (the “parent”). Finally, the hierarchy network was styled, communities were labeled by functional enrichment using gProfiler (via CDAPS), p values were calculated based on the accumulative hypergeometric distribution, and a layout was applied. The STRING—Human Protein Links—High Confidence (Score ≥ 0.7) network is available in the Network Data Exchange (NDEx) at https://www.ndexbio.org/viewer/networks/275bd84e-3d18-11e8-a935-0ac135e8bacf.

### Generation pseudotyped SARS-CoV-2 virus

VSV pseudotyped with Spike (S) protein of SARS-CoV-2 wild-type (Wuhan-Hu-1) were generated according to a published protocol [70]. Briefly, BHK-21/WI-2 cells (Kerafast, MA) transfected with SARS-CoV-2 S protein were inoculated with VSV-G pseudotyped ΔG-luciferase VSV (Kerafast, MA). After a 2 h incubation at 37 °C, the inoculum was removed and cells were treated with DMEM supplemented with 5% FBS, 50 U/mL penicillin, and 50 µg/mL streptomycin. Pseudotyped particles were collected 24 h post-inoculation, then centrifuged at 1,000*g* to remove cell debris and stored at −80 °C until use.

### Mapping factors into the SARS-CoV-2 replication cycle

Caco-2 cells were transfected with indicated siRNAs and incubated for 48 h at 37°C, 5% CO_2_. To determine the effect of the identified factors on viral entry, cells were infected with VSV-S-luciferase or VSV-G-luciferase and incubated for 16 h. The activity of firefly luciferase was then quantified using the bright-Glo luciferase assay (Promega). To measure RNA replication and late stages, cells were infected with SARS-CoV-2 (USA-WA1/2020) at a MOI 5 for 1 h on ice. Viral inoculum was removed and cells were washed twice with 1×PBS and supplemented with cell growth media. At 6 h post-infection, intracellular viral RNA was purified from infected cells using the TurboCapture mRNA Kit (Qiagen) in accordance with the manufacturer’s instructions. The purified RNA was subjected to first-strand cDNA synthesis using the high-capacity cDNA reverse transcription kit (Applied Biosystems). Real-time quantitative PCR (RT-qPCR) analysis was then performed using TaqPath one-step RT-qPCR Master Mix (Applied Biosystems) and ActinB CTRL Mix (Applied Biosystems) for housekeeping genes, and the following primers and probe for qPCR measurements of viral genes: N-Fwd: 5′-TTACAAACATTGGCCGCAAA-3′; N-Rev: 5′-GCGCGACATTCCGAAGAA-3′; N-Probe: 5′-FAM-ACAATTTGCCCCCAGCGCTTCAG-BHQ-3′. To evaluate late stages, supernatants collected at 18 h post-infection were used to infect naïve Vero E6 cells. At 18 h post-infection, cells were fixed with 5% PFA (Boston BioProducts) for 4 h at room temperature and then subjected to immunostaining and imaging for SARS-CoV-2 N protein.

### Binding of Spike protein to Perlecan

Immunopurified Perlecan isolated from human coronary artery endothelial cells [38] (10 µg/mL in Dulbecco’s phosphate-buffered saline (DPBS) pH 7.4) was immobilized onto gold sensor chips (Sensor chip Au, Cytiva) at 5 µL/min in an SPR system (Biacore T200, Cytiva) at 25 °C for 240s. The sensor chip flow channels were then washed with DPBS at 5 μL/min until a stable response unit (RU) was achieved. The flow channels were then exposed to bovine serum albumin (BSA; 10 mg/mL in DPBS) at a flow rate of 5 μL/min for 240s and washed with DPBS until a stable RU was observed. Control flow channels contained immobilized BSA. Spike protein (25, 50, 100 and 200 nM in DPBS) was exposed to the flow channels at a flow rate of 10 μL/min for 120s. The dissociation of Spike protein was measured in the following 600s. The RU values throughout the experiment for BSA were subtracted from the RU values for Perlecan to determine the level of specific binding. This experiment was repeated with Perlecan treated with heparinase III (0.01 U/mL in DPBS for 16 h at 37 °C; EC 4.2.2.8; Iduron, Cheshire, UK) to remove HS. n = 3 per condition.

### Evaluation of host factors using SARS-CoV-1, MERS-CoV and OC43-CoV

A549 cells stably expressing DPP4 or ACE2 were subject to siRNA mediated knockdown of select host factors for 72 hours prior to use. Transfection was performed as described in [71], modified for a 96 well plate format. Briefly, 0.7µl Opti-MEM (Gibco) was mixed with 0.35µl Oligofectamine (Thermo Scientific) and incubated for 5 min at room temperature (RT) and then mixed with 6µl Opti-MEM and 4µl of 0.50µM siRNA. This mix was incubated for 20 min at RT. A further 45µl of Opti-MEM was added to the transfection mixture, media were removed from cells and 50µl of transfection mixture was added. After a 4h incubation at 37°C/5% CO2, 50µl of 20% FBS DMEM was added to the cells and incubated at 37°C/5% CO2, for 3 days prior to experimental use. A549-DPP4 cells were infected with MERS-CoV (Jordan strain) and A549-ACE2 cells were infected with SARS-CoV (MA15 strain), both at MOI 0.1. 48-hour post infection, supernatant from infected cells was collected and virus titer determined by TCID50 assay (as described [72]). Two experiments were performed and the average TCID50/ml calculated. Scrambled siRNA sequences acted as a negative control and ACE2 and DPP4 targeting siRNAs were positive controls.

### Inhibition of SARS-CoV-2 replication in vitro by Smac mimetics

Caco-2 cells were treated with the compounds (AZD5582 and SBI-0953294) for 18h prior to infection with SARS-CoV-2 (Wuhan-1 isolate) at MOI of 0.625. Forty-eight hours post-infection, the infected cells were fixed with 4% paraformaldehyde for 2 h and permeabilized with 0.5% Triton X-100 for 15 min. After blocking with 3% bovine serum albumin (BSA) for 15 min, cells were incubated with rabbit anti-SARS-CoV-2 NP antibodies for 1hours. After two washes with phosphate-buffered saline (PBS), the cells were incubated with Alexa Fluor 488-conjugated goat-anti-rabbit IgG (Thermo Fisher Scientific) for 1 h at room temperature. After two additional washes, the cells were mounted with DAPI (BioLegend) and images were acquired using the Celigo Image Cytometer (Nexcelom).

### In vivo experiments

Male K18-hACE2 mice, aged 6−10 weeks old, were kept in biosafety level housing and given access to standard pellet feed and water ad libitum as we previously described. Mice were randomly allocated to experimental groups (*n* = 3 for Omicron experiment, *n* = 11 for Alpha experiment) for antiviral evaluation. The experiments were not blinded. Experimentally, each mouse was intranasally inoculated with 10,000 PFU of SARS-CoV-2 (Omicron BA.5) or 200 PFU (Alpha B.1.1.7) in 20 µL PBS under intraperitoneal ketamine and xylazine anesthesia. Twelve hours before-virus-challenge, mice were intraperitoneally given either Nirmatrelvir (200 mg/kg), or AZD5582 (3 mg/kg) or 1% DMSO in PBS (vehicle control). The second and third doses of drug treatment was performed at 12 and 36 hpi, respectively. For Omicron experiments, three animals in each group were sacrificed at 3 dpi for virological analyses (Omicron). Lung tissue samples were collected. Viral yield in the tissue homogenates were detected by plaque assay. For Alpha experiments, animals (*n* = 5) were monitored twice daily for clinical signs of disease. Their body weight and survival were monitored for 14 days or until death. Six animals in each group were sacrificed at 3 dpi for virological analyses. Lung tissue samples were collected. Viral yield in the tissue homogenates were detected by plaque assay. A 30% body weight loss is set as human endpoint.

## Statistics

Statistical parameters including the exact value of n, dispersion, and precision measures (mean ± SD or SEM), and statistical significance are reported in the figures and figure legends. Statistical significance between groups was determined using GraphPad Prism v8.0 (GraphPad, San Diego, CA), and the test used is indicated in the figure legends.

## Supporting information

S1 Fig Genome-wide siRNA screen identifies host factors involved in SARS-CoV-2 replication. **(A)** Dot plot shows average SARS-CoV-2 infectivity Z-score values from the genome-wide siRNA screen. Controls are shown (non-targeting scrambled siRNA, negative; siACE2 and siTMPRSS2, positive). **(B)** Correlation plots of Z-score values for genome-wide siRNA screens using Caco-2 cells infected with SARS-CoV-2. *R* = Pearson correlation coefficient between screens. **(C)** Visualization of Gene Set Overlap Across Genetic Screens. The Circos plot illustrates the overlap of gene sets identified by the different genetic screens. Each segment of the outer circle represents a dataset, and its width reflects the total number of unique genes identified in the corresponding screen. Purple arcs connect segments to indicate shared genes (intersections) between datasets, with the density and intensity of connections reflecting the magnitude of overlap. This plot highlights the relationships and distinctiveness among the genetic screens, with some datasets exhibiting sparse connectivity (low overlap), suggesting a higher degree of uniqueness in their identified gene sets. The data underlying this figure can be found in S1 Data.
(TIFF)

S2 Fig Expression of the identified host factors in SARS-CoV-2 target cells. Heatmap shows percentage of detectable levels of expression of a given factor in the indicated cell type [74]. % expression >1 was considered a detectable level. The data underlying this figure can be found in S1 Data.
(TIFF)

S3 Fig Network integration reveals relevant networks implicated in SARS-CoV-2 replication. The network containing the identified proviral (**green**) and antiviral (**red**) human host factors was integrated with host factors reported to be relevant for SARS-CoV-2 infection. These include genetic CRISPR screen hits (Wei and colleagues, 2020 [15], **light pink**; Daniloski and colleagues, 2020 [16], **dark pink**), protein–protein interaction hits (Stukalov and colleagues, 2020 [31], **blue**; Gordon and colleagues, 2020 [32], **purple**), as well as hits from a phosphoproteomics study (Bouhaddou and colleagues, 2020 [33], **yellow**). The network was subjected to supervised community detection [67,73], and the resultant hierarchy is shown. Each node represents a cluster of densely interconnected proteins, and each edge (arrow) denotes containment of one community (edge target) by another (edge source). Labels indicate enriched biological processes. The percentage of each community that corresponds to each dataset is shown by matching colors. Edges indicate interactions from STRING database. * indicates highlighted clusters shown in Fig 2.
(TIFF)

S4 Fig Mapping of host factors into SARS-CoV-2 infectious cycle. **(A)** Caco-2 cells subjected to siRNA-mediated knockdown of the indicated host factors were infected with SARS-CoV-2 pseudotyped VSV luciferase virus (VSV-S) or VSV luciferase virus expressing its natural glycoprotein (VSV-G) for 18h prior to measurement of luciferase signal. Data represent mean from one representative experiment in duplicate (*n* = 2). **(B,C)** Binding of spike protein and RBD to perlecan. Surface plasmon resonance (SPR) was used to evaluate spike binding to perlecan. This experiment was repeated twice. **(D)** Surface plasmon resonance (SPR) was used to evaluate binding of RBD to perlecan or perlecan without HS spike binding to immunopurified perlecan isolated from human coronary artery endothelial cells. Control flow channels contained immobilized BSA. RBD at indicated concentrations was run across the flow channels for 120 s and dissociation was measured in the following 600 s. The RU values throughout the experiment for BSA were subtracted from the RU values for perlecan to determine the level of specific binding. This experiment was repeated with perlecan treated with heparinase III. The data underlying this figure can be found in S1 Data.
(TIFF)

S5 Fig Pharmacological inhibition of BIRC2 reduces SARS-CoV-2 replication in vitro and in vivo. **(A)** Cells were treated with AZD5582 at the indicated concentrations. Twenty-four hours post-treatment, the cell lysates were analyzed by Western blotting for p100/p52 protein. A representative immunoblot presented here demonstrate that AZD5582 treatment induces the cleavage of p100. **(B)** Layout of mice experiments using SARS-CoV-2 B.1.1.7 (Alpha) infection. Effect of AZD5582 on SARS-CoV-2 replication in survival **(C)** and body weight **(D)** were recorded for 14 days post-infection. Virus titer as measured in the lungs of infected mice by plaque assay **(E)** were performed on 3 dpi. Tissue sampling was done at 72 hpi. One-way ANOVA when compared with the vehicle control group. *P < 0.05, ****P < 0.001. The data underlying this figure can be found in S1 Data. The original uncropped blots can be found in S1 Raw Images.
(TIFF)

S1 Table Genome-wide siRNA screen data. Tables include the raw and processed data for the genome-wide siRNA data, as well as the list of enriched proviral and antiviral gene ontology (GO) terms.
(XLSX)

S2 Table Unique host factors. List of host factors identified in this study but not in previous CRISPR screens as listed in Grodzki and colleagues 2022 [22].
(XLSX)

S3 Table Validated host factors. List of host factors that were confirmed to affect the replication of SARS-CoV-2 with two or more siRNAs.
(XLSX)

S4 Table Overlap with previous screens. List of host factors mapping to discrete stages of SARS-CoV-2 infectious cycle and were identified by previous functional genetic screens.
(XLSX)

S1 Raw Images Uncropped western blots related to Figs 4B and S5A.
(PDF)

S1 Data (XLSX)

## Abbreviations

**:**
BIRC2: Baculoviral IAP Repeat Containing 2
CDAPS: Community Detection Application and Service
DPBS: Dulbecco’s phosphate-buffered saline
GO: gene ontology
HS: Heparan sulfate
IAPs: inhibitor of apoptosis proteins
PBS: phosphate-buffered saline
PFU: plaque forming unit
PPIs: protein–protein interactions
RBD: receptor binding domain
RT-qPCR: real-time quantitative PCR
SARS-CoV-2: severe acute respiratory syndrome coronavirus 2
VSV: vesicular stomatitis virus

## References

[1] Zhu Z, Lian X, Su X, Wu W, Marraro GA, Zeng Y. From SARS and MERS to COVID-19: a brief summary and comparison of severe acute respiratory infections caused by three highly pathogenic human coronaviruses. Respir Res. 2020;21(1):224. doi: 10.1186/s12931-020-01479-w 32854739 PMC7450684

[2] Abdelrahman Z, Li M, Wang X. Comparative review of SARS-CoV-2, SARS-CoV, MERS-CoV, and influenza A respiratory viruses. Front Immunol. 2020;11:552909. doi: 10.3389/fimmu.2020.552909 33013925 PMC7516028

[3] Carabelli AM, Peacock TP, Thorne LG, Harvey WT, Hughes J, COVID-19 Genomics UK Consortium, et al. SARS-CoV-2 variant biology: immune escape, transmission and fitness. Nat Rev Microbiol. 2023;21(3):162–77. doi: 10.1038/s41579-022-00841-7 36653446 PMC9847462

[4] The evolution of SARS-CoV-2 | Nature Reviews Microbiology https://www.nature.com/articles/s41579-023-00878-2. 10.1038/s41579-023-00878-2 37020110

[5] Kaufmann SHE, Dorhoi A, Hotchkiss RS, Bartenschlager R. Host-directed therapies for bacterial and viral infections. Nat Rev Drug Discov. 2018;17(1):35–56. doi: 10.1038/nrd.2017.162 28935918 PMC7097079

[6] Host-directed therapies for infectious diseases: current status, recent progress, and future prospects—PMC https://www.ncbi.nlm.nih.gov/pmc/articles/PMC7164794/. 10.1016/S1473-3099(16)00078-5 PMC7164794 27036359

[7] Zhou P, Yang X-L, Wang X-G, Hu B, Zhang L, Zhang W, et al. Discovery of a novel coronavirus associated with the recent pneumonia outbreak in humans and its potential bat origin. Cold Spring Harbor Laboratory. 2020. doi: 10.1101/2020.01.22.914952

[8] Jelinek HF, Mousa M, Alefishat E, Osman W, Spence I, Bu D, et al. Evolution, ecology, and zoonotic transmission of betacoronaviruses: a review. Front Vet Sci. 2021;8:644414. doi: 10.3389/fvets.2021.644414 34095271 PMC8173069

[9] Mechanisms of SARS-CoV-2 entry into cells | Nature Reviews Molecular Cell Biology https://www.nature.com/articles/s41580-021-00418-x. 10.1038/s41580-021-00418-x PMC8491763 34611326

[10] Fehr AR, Perlman S. Coronaviruses: an overview of their replication and pathogenesis. Methods Mol Biol. 2015;1282:1–23. doi: 10.1007/978-1-4939-2438-7_1 25720466 PMC4369385

[11] Thorne LG, Bouhaddou M, Reuschl A-K, Zuliani-Alvarez L, Polacco B, Pelin A, et al. Evolution of enhanced innate immune evasion by SARS-CoV-2. Nature. 2022;602(7897):487–95. doi: 10.1038/s41586-021-04352-y 34942634 PMC8850198

[12] Miorin L, Kehrer T, Sanchez-Aparicio MT, Zhang K, Cohen P, Patel RS, et al. SARS-CoV-2 Orf6 hijacks Nup98 to block STAT nuclear import and antagonize interferon signaling. Proc Natl Acad Sci U S A. 2020;117(45):28344–54. doi: 10.1073/pnas.2016650117 33097660 PMC7668094

[13] Hagemeijer MC, Verheije MH, Ulasli M, Shaltiël IA, de Vries LA, Reggiori F, et al. Dynamics of coronavirus replication-transcription complexes. J Virol. 2010;84(4):2134–49. doi: 10.1128/JVI.01716-09 20007278 PMC2812403

[14] Hoffmann M, Kleine-Weber H, Schroeder S, Krüger N, Herrler T, Erichsen S, et al. SARS-CoV-2 cell entry depends on ACE2 and TMPRSS2 and is blocked by a clinically proven protease inhibitor. Cell. 2020;181(2):271-280.e8. doi: 10.1016/j.cell.2020.02.052 32142651 PMC7102627

[15] Wei J, Alfajaro MM, DeWeirdt PC, Hanna RE, Lu-Culligan WJ, Cai WL, et al. Genome-wide CRISPR screens reveal host factors critical for SARS-CoV-2 infection. Cell. 2021;184(1):76-91.e13. doi: 10.1016/j.cell.2020.10.028 33147444 PMC7574718

[16] Daniloski Z, Jordan TX, Wessels H-H, Hoagland DA, Kasela S, Legut M, et al. Identification of required host factors for SARS-CoV-2 infection in human cells. Cell. 2021;184(1):92-105.e16. doi: 10.1016/j.cell.2020.10.030 33147445 PMC7584921

[17] Schneider WM, Luna JM, Hoffmann H-H, Sánchez-Rivera FJ, Leal AA, Ashbrook AW, et al. Genome-scale identification of SARS-CoV-2 and pan-coronavirus host factor networks. Cell. 2021;184(1):120-132.e14. doi: 10.1016/j.cell.2020.12.006 33382968 PMC7796900

[18] Wang R, Simoneau CR, Kulsuptrakul J, Bouhaddou M, Travisano KA, Hayashi JM, et al. Genetic screens identify host factors for SARS-CoV-2 and common cold coronaviruses. Cell. 2021;184(1):106-119.e14. doi: 10.1016/j.cell.2020.12.004 33333024 PMC7723770

[19] Kratzel A, Kelly JN, V’kovski P, Portmann J, Brüggemann Y, Todt D, et al. A genome-wide CRISPR screen identifies interactors of the autophagy pathway as conserved coronavirus targets. PLoS Biol. 2021;19(12):e3001490. doi: 10.1371/journal.pbio.3001490 34962926 PMC8741300

[20] Baggen J, Persoons L, Vanstreels E, Jansen S, Van Looveren D, Boeckx B, et al. Genome-wide CRISPR screening identifies TMEM106B as a proviral host factor for SARS-CoV-2. Nat Genet. 2021;53(4):435–44. doi: 10.1038/s41588-021-00805-2 33686287

[21] Rebendenne A, Roy P, Bonaventure B, Chaves Valadão AL, Desmarets L, Arnaud-Arnould M, et al. Bidirectional genome-wide CRISPR screens reveal host factors regulating SARS-CoV-2, MERS-CoV and seasonal HCoVs. Nat Genet. 2022;54(8):1090–102. doi: 10.1038/s41588-022-01110-2 35879413 PMC11627114

[22] Grodzki M, Bluhm AP, Schaefer M, Tagmount A, Russo M, Sobh A, et al. Genome-scale CRISPR screens identify host factors that promote human coronavirus infection. Genome Med. 2022;14(1):10. doi: 10.1186/s13073-022-01013-1 35086559 PMC8792531

[23] Hoffmann H-H, Schneider WM, Rozen-Gagnon K, Miles LA, Schuster F, Razooky B, et al. TMEM41B is a pan-flavivirus host factor. Cell. 2021;184(1):133-148.e20. doi: 10.1016/j.cell.2020.12.005 33338421 PMC7954666

[24] Zhu Y, Feng F, Hu G, Wang Y, Yu Y, Zhu Y, et al. A genome-wide CRISPR screen identifies host factors that regulate SARS-CoV-2 entry. Nat Commun. 2021;12(1):961. doi: 10.1038/s41467-021-21213-4 33574281 PMC7878750

[25] Chan K, Farias AG, Lee H, Guvenc F, Mero P, Brown KR, et al. Survival-based CRISPR genetic screens across a panel of permissive cell lines identify common and cell-specific SARS-CoV-2 host factors. Heliyon. 2023;9(1):e12744. doi: 10.1016/j.heliyon.2022.e12744 36597481 PMC9800021

[26] Le Pen J, Paniccia G, Kinast V, Moncada-Velez M, Ashbrook AW, Bauer M, et al. A genome-wide arrayed CRISPR screen identifies PLSCR1 as an intrinsic barrier to SARS-CoV-2 entry that recent virus variants have evolved to resist. PLoS Biol. 2024;22(9):e3002767. doi: 10.1371/journal.pbio.3002767 39316623 PMC11486371

[27] Gupta A, Madhavan MV, Sehgal K, Nair N, Mahajan S, Sehrawat TS, et al. Extrapulmonary manifestations of COVID-19. Nat Med. 2020;26(7):1017–32. doi: 10.1038/s41591-020-0968-3 32651579 PMC11972613

[28] Lamers MM, Beumer J, van der Vaart J, Knoops K, Puschhof J, Breugem TI, et al. SARS-CoV-2 productively infects human gut enterocytes. Science. 2020;369(6499):50–4. doi: 10.1126/science.abc1669 32358202 PMC7199907

[29] Martin-Sancho L, Lewinski MK, Pache L, Stoneham CA, Yin X, Becker ME, et al. Functional landscape of SARS-CoV-2 cellular restriction. Mol Cell. 2021;81(12):2656-2668.e8. doi: 10.1016/j.molcel.2021.04.008 PMC8043580 33930332

[30] Sungnak W, Huang N, Bécavin C, Berg M, Queen R, Litvinukova M, et al. SARS-CoV-2 entry factors are highly expressed in nasal epithelial cells together with innate immune genes. Nat Med. 2020;26(5):681–7. doi: 10.1038/s41591-020-0868-6 32327758 PMC8637938

[31] Stukalov A, Girault V, Grass V, Karayel O, Bergant V, Urban C, et al. Multilevel proteomics reveals host perturbations by SARS-CoV-2 and SARS-CoV. Nature. 2021;594(7862):246–52. doi: 10.1038/s41586-021-03493-4 33845483

[32] Gordon DE, Jang GM, Bouhaddou M, Xu J, Obernier K, White KM, et al. A SARS-CoV-2 protein interaction map reveals targets for drug repurposing. Nature. 2020;583(7816):459–68. doi: 10.1038/s41586-020-2286-9 32353859 PMC7431030

[33] Bouhaddou M, Memon D, Meyer B, White KM, Rezelj VV, Correa Marrero M, et al. The global phosphorylation landscape of SARS-CoV-2 infection. Cell. 2020;182(3):685-712.e19. doi: 10.1016/j.cell.2020.06.034 32645325 PMC7321036

[34] A Crisp(r) New Perspective on SARS-CoV-2 Biology—ScienceDirect. Available from: https://www.sciencedirect.com/science/article/pii/S0092867420316251?ref=pdf_download&fr=RR-2&rr=809284161b317792#bib5 10.1016/j.cell.2020.12.003 PMC7746090 33338422

[35] Farach-Carson MC, Carson DD. Perlecan—a multifunctional extracellular proteoglycan scaffold. Glycobiology. 2007;17(9):897–905. doi: 10.1093/glycob/cwm043 17442708

[36] Cagno V, Tseligka ED, Jones ST, Tapparel C. Heparan sulfate proteoglycans and viral attachment: true receptors or adaptation bias?. Viruses. 2019;11(7):596. doi: 10.3390/v11070596 31266258 PMC6669472

[37] Clausen TM, Sandoval DR, Spliid CB, Pihl J, Perrett HR, Painter CD, et al. SARS-CoV-2 infection depends on cellular heparan sulfate and ACE2. Cell. 2020;183(4):1043-1057.e15. doi: 10.1016/j.cell.2020.09.033 32970989 PMC7489987

[38] Whitelock JM, Graham LD, Melrose J, Murdoch AD, Iozzo RV, Underwood PA. Human perlecan immunopurified from different endothelial cell sources has different adhesive properties for vascular cells. Matrix Biol. 1999;18(2):163–78. doi: 10.1016/s0945-053x(99)00014-1 10372557

[39] V’kovski P, Kratzel A, Steiner S, Stalder H, Thiel V. Coronavirus biology and replication: implications for SARS-CoV-2. Nat Rev Microbiol. 2021;19(3):155–70. doi: 10.1038/s41579-020-00468-6 33116300 PMC7592455

[40] Ghosh S, Dellibovi-Ragheb TA, Kerviel A, Pak E, Qiu Q, Fisher M, et al. β-Coronaviruses use lysosomes for egress instead of the biosynthetic secretory pathway. Cell. 2020;183(6):1520-1535.e14. doi: 10.1016/j.cell.2020.10.039 33157038 PMC7590812

[41] Marazzi I, Ho JSY, Kim J, Manicassamy B, Dewell S, Albrecht RA, et al. Suppression of the antiviral response by an influenza histone mimic. Nature. 2012;483(7390):428–33. doi: 10.1038/nature10892 22419161 PMC3598589

[42] Li D, Liu Y, Lu Y, Gao S, Zhang L. Palmitoylation of SARS-CoV-2 S protein is critical for S-mediated syncytia formation and virus entry. J Med Virol. 2022;94(1):342–8. doi: 10.1002/jmv.27339 34528721 PMC8661603

[43] Stuchell MD, Garrus JE, Müller B, Stray KM, Ghaffarian S, McKinnon R, et al. The human endosomal sorting complex required for transport (ESCRT-I) and its role in HIV-1 budding. J Biol Chem. 2004;279(34):36059–71. doi: 10.1074/jbc.M405226200 15218037

[44] Sato T, Suzuki T, Watanabe H, Kadowaki A, Fukamizu A, Liu PP, et al. Apelin is a positive regulator of ACE2 in failing hearts. J Clin Invest. 2013;123(12):5203–11. doi: 10.1172/JCI69608 24177423 PMC3859384

[45] Pache L, Dutra MS, Spivak AM, Marlett JM, Murry JP, Hwang Y, et al. BIRC2/cIAP1 is a negative regulator of HIV-1 transcription and can be targeted by smac mimetics to promote reversal of viral latency. Cell Host Microbe. 2015;18(3):345–53. doi: 10.1016/j.chom.2015.08.009 26355217 PMC4617541

[46] Pache L, Marsden MD, Teriete P, Portillo AJ, Heimann D, Kim JT, Soliman MSA, Dimapasoc M, Carmona C, Celeridad M, et al. Pharmacological activation of non-canonical NF-κB signaling activates latent HIV-1 reservoirs in vivo. Cell Rep Med. 2020;1:100037. doi: 10.1016/j.xcrm.2020.100037 PMC7659604 33205060

[47] Nixon CC, Mavigner M, Sampey GC, Brooks AD, Spagnuolo RA, Irlbeck DM, et al. Systemic HIV and SIV latency reversal via non-canonical NF-κB signalling in vivo. Nature. 2020;578(7793):160–5. doi: 10.1038/s41586-020-1951-3 31969707 PMC7111210

[48] Zarnegar BJ, Wang Y, Mahoney DJ, Dempsey PW, Cheung HH, He J, et al. Noncanonical NF-κB activation requires coordinated assembly of a regulatory complex of the adaptors cIAP1, cIAP2, TRAF2 and TRAF3 and the kinase NIK. Nat Immunol. 2008;9(12):1371–8. doi: 10.1038/ni.1676 18997794 PMC2676931

[49] Mei M, Impagnatiello MA, Jiao J, Reiser U, Tontsch-Grunt U, Zhang J, et al. An orally-available monovalent SMAC mimetic compound as a broad-spectrum antiviral. Protein Cell. 2024;15(1):69–75. doi: 10.1093/procel/pwad033 37294910 PMC10762662

[50] Emanuel W, Kirstin M, Vedran F, Asija D, Theresa GL, Roberto A, Filippos K, David K, Salah A, Christopher B, et al. Bulk and single-cell gene expression profiling of SARS-CoV-2 infected human cell lines identifies molecular targets for therapeutic intervention (Systems Biology). 2020. doi: 10.1101/2020.05.05.079194

[51] Xu Z, Elaish M, Wong CP, Hassan BB, Lopez-Orozco J, Felix-Lopez A, et al. The Wnt/β-catenin pathway is important for replication of SARS-CoV-2 and other pathogenic RNA viruses. Npj Viruses. 2024;2(1):6. doi: 10.1038/s44298-024-00018-4 40295745 PMC11721380

[52] Gong Y, Qin S, Dai L, Tian Z. The glycosylation in SARS-CoV-2 and its receptor ACE2. Signal Transduct Target Ther. 2021;6(1):396. doi: 10.1038/s41392-021-00809-8 34782609 PMC8591162

[53] Higgins CA, Nilsson-Payant BE, Bonaventure B, Kurland AP, Ye C, Yaron TM, et al. SARS-CoV-2 hijacks p38β/MAPK11 to promote virus replication. mBio. 2023;14(4):e0100723. doi: 10.1128/mbio.01007-23 37345956 PMC10470746

[54] Lloyd RE. Nuclear proteins hijacked by mammalian cytoplasmic plus strand RNA viruses. Virology. 2015;479–480:457–74. doi: 10.1016/j.virol.2015.03.001 25818028 PMC4426963

[55] Netsawang J, Noisakran S, Puttikhunt C, Kasinrerk W, Wongwiwat W, Malasit P, et al. Nuclear localization of dengue virus capsid protein is required for DAXX interaction and apoptosis. Virus Res. 2010;147(2):275–83. doi: 10.1016/j.virusres.2009.11.012 19944121

[56] Cohen S, Au S, Panté N. How viruses access the nucleus. Biochim Biophys Acta. 2011;1813(9):1634–45. doi: 10.1016/j.bbamcr.2010.12.009 21167871

[57] Bouhaddou M, Reuschl A-K, Polacco BJ, Thorne LG, Ummadi MR, Ye C, et al. SARS-CoV-2 variants evolve convergent strategies to remodel the host response. Cell. 2023;186(21):4597-4614.e26. doi: 10.1016/j.cell.2023.08.026 37738970 PMC10604369

[58] Zhang Q, Chen CZ, Swaroop M, Xu M, Wang L, Lee J, et al. Heparan sulfate assists SARS-CoV-2 in cell entry and can be targeted by approved drugs in vitro. Cell Discov. 2020;6(1):80. doi: 10.1038/s41421-020-00222-5 33298900 PMC7610239

[59] Ling J, Li J, Khan A, Lundkvist Å, Li J-P. Is heparan sulfate a target for inhibition of RNA virus infection?. Am J Physiol Cell Physiol. 2022;322(4):C605–13. doi: 10.1152/ajpcell.00028.2022 35196165 PMC8977144

[60] Silke J, Meier P. Inhibitor of apoptosis (IAP) proteins-modulators of cell death and inflammation. Cold Spring Harb Perspect Biol. 2013;5(2):a008730. doi: 10.1101/cshperspect.a008730 23378585 PMC3552501

[61] Bai L, Smith DC, Wang S. Small-molecule SMAC mimetics as new cancer therapeutics. Pharmacol Ther. 2014;144(1):82–95. doi: 10.1016/j.pharmthera.2014.05.007 24841289 PMC4247261

[62] Roberts A, Deming D, Paddock CD, Cheng A, Yount B, Vogel L, et al. A mouse-adapted SARS-coronavirus causes disease and mortality in BALB/c mice. PLoS Pathog. 2007;3(1):e5. doi: 10.1371/journal.ppat.0030005 17222058 PMC1769406

[63] Hultquist JF, Hiatt J, Schumann K, McGregor MJ, Roth TL, Haas P, et al. CRISPR-Cas9 genome engineering of primary CD4+ T cells for the interrogation of HIV-host factor interactions. Nat Protoc. 2019;14(1):1–27. doi: 10.1038/s41596-018-0069-7 30559373 PMC6637941

[64] Kramer M, Dutkowski J, Yu M, Bafna V, Ideker T. Inferring gene ontologies from pairwise similarity data. Bioinformatics. 2014;30(12):i34-42. doi: 10.1093/bioinformatics/btu282 24932003 PMC4058954

[65] Yu MK, Kramer M, Dutkowski J, Srivas R, Licon K, Kreisberg J, et al. Translation of genotype to phenotype by a hierarchy of cell subsystems. Cell Syst. 2016;2(2):77–88. doi: 10.1016/j.cels.2016.02.003 26949740 PMC4772745

[66] Dutkowski J, Kramer M, Surma MA, Balakrishnan R, Cherry JM, Krogan NJ, et al. A gene ontology inferred from molecular networks. Nat Biotechnol. 2013;31(1):38–45. doi: 10.1038/nbt.2463 23242164 PMC3654867

[67] Carlin DE, Demchak B, Pratt D, Sage E, Ideker T. Network propagation in the cytoscape cyberinfrastructure. PLoS Comput Biol. 2017;13(10):e1005598. doi: 10.1371/journal.pcbi.1005598 29023449 PMC5638226

[68] Singhal A, Cao S, Churas C, Pratt D, Fortunato S, Zheng F, et al. Multiscale community detection in Cytoscape. PLoS Comput Biol. 2020;16(10):e1008239. doi: 10.1371/journal.pcbi.1008239 33095781 PMC7584444

[69] Zheng F, Zhang S, Churas C, Pratt D, Bahar I, Ideker T. HiDeF: identifying persistent structures in multiscale ’omics data. Genome Biol. 2021;22(1):21. doi: 10.1186/s13059-020-02228-4 33413539 PMC7789082

[70] Whitt MA. Generation of VSV pseudotypes using recombinant ΔG-VSV for studies on virus entry, identification of entry inhibitors, and immune responses to vaccines. J Virol Methods. 2010;169(2):365–74. doi: 10.1016/j.jviromet.2010.08.006 20709108 PMC2956192

[71] Weston S, Baracco L, Keller C, Matthews K, McGrath ME, Logue J, et al. The SKI complex is a broad-spectrum, host-directed antiviral drug target for coronaviruses, influenza, and filoviruses. Proc Natl Acad Sci U S A. 2020;117(48):30687–98. doi: 10.1073/pnas.2012939117 33184176 PMC7720140

[72] Coleman CM, Frieman MB. Growth and quantification of MERS-CoV infection. Curr Protoc Microbiol. 2015;37(1):15E.2.1-9. doi: 10.1002/9780471729259.mc15e02s37 26344219 PMC4735735

[73] Shannon P, Markiel A, Ozier O, Baliga NS, Wang JT, Ramage D, et al. Cytoscape: a software environment for integrated models of biomolecular interaction networks. Genome Res. 2003;13(11):2498–504. doi: 10.1101/gr.1239303 14597658 PMC403769

[74] Deprez M, Zaragosi L-E, Truchi M, Becavin C, Ruiz García S, Arguel M-J, et al. A single-cell atlas of the human healthy airways. Am J Respir Crit Care Med. 2020;202(12):1636–45. doi: 10.1164/rccm.201911-2199OC 32726565

