# Supplementary figures and images for: Global siRNA screen identifies human host factors critical for SARS-CoV-2 replication and late stages of infection
Source: PLoS Biol. 2025 Jun 12;23(6):e3002738. doi: 10.1371/journal.pbio.3002738 (PMC12204624; doi:10.1371/journal.pbio.3002738)

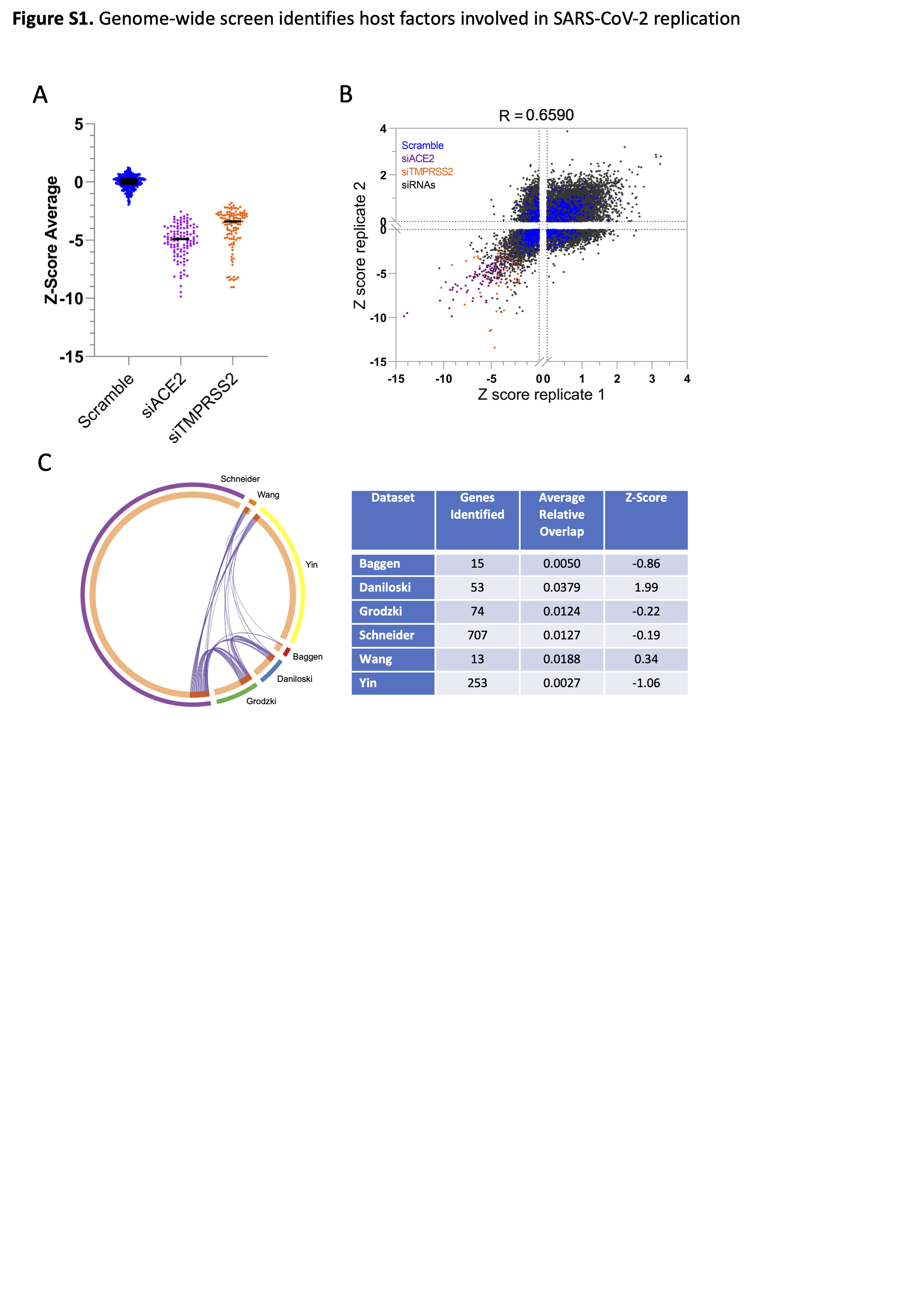

Supplement: S1 Fig — (A) Dot plot shows average SARS-CoV-2 infectivity Z-score values from the genome-wide siRNA screen. Controls are shown (non-targeting scrambled siRNA, negative; siACE2 and siTMPRSS2, positive). (B) Correlation plots of Z-score values for genome-wide siRNA screens using Caco-2 cells infected with SARS-CoV-2. R = Pearson correlation coefficient between screens. (C) Visualization of Gene Set Overlap Across Genetic Screens. The Circos plot illustrates the overlap of gene sets identified by the different genetic screens. Each segment of the outer circle represents a dataset, and its width reflects the total number of unique genes identified in the corresponding screen. Purple arcs connect segments to indicate shared genes (intersections) between datasets, with the density and intensity of connections reflecting the magnitude of overlap. This plot highlights the relationships and distinctiveness among the genetic screens, with some datasets exhibiting sparse connectivity (low overlap), suggesting a higher degree of uniqueness in their identified gene sets. The data underlying this figure can be found in S1 Data. (TIFF) [file pbio.3002738.s001.tiff]

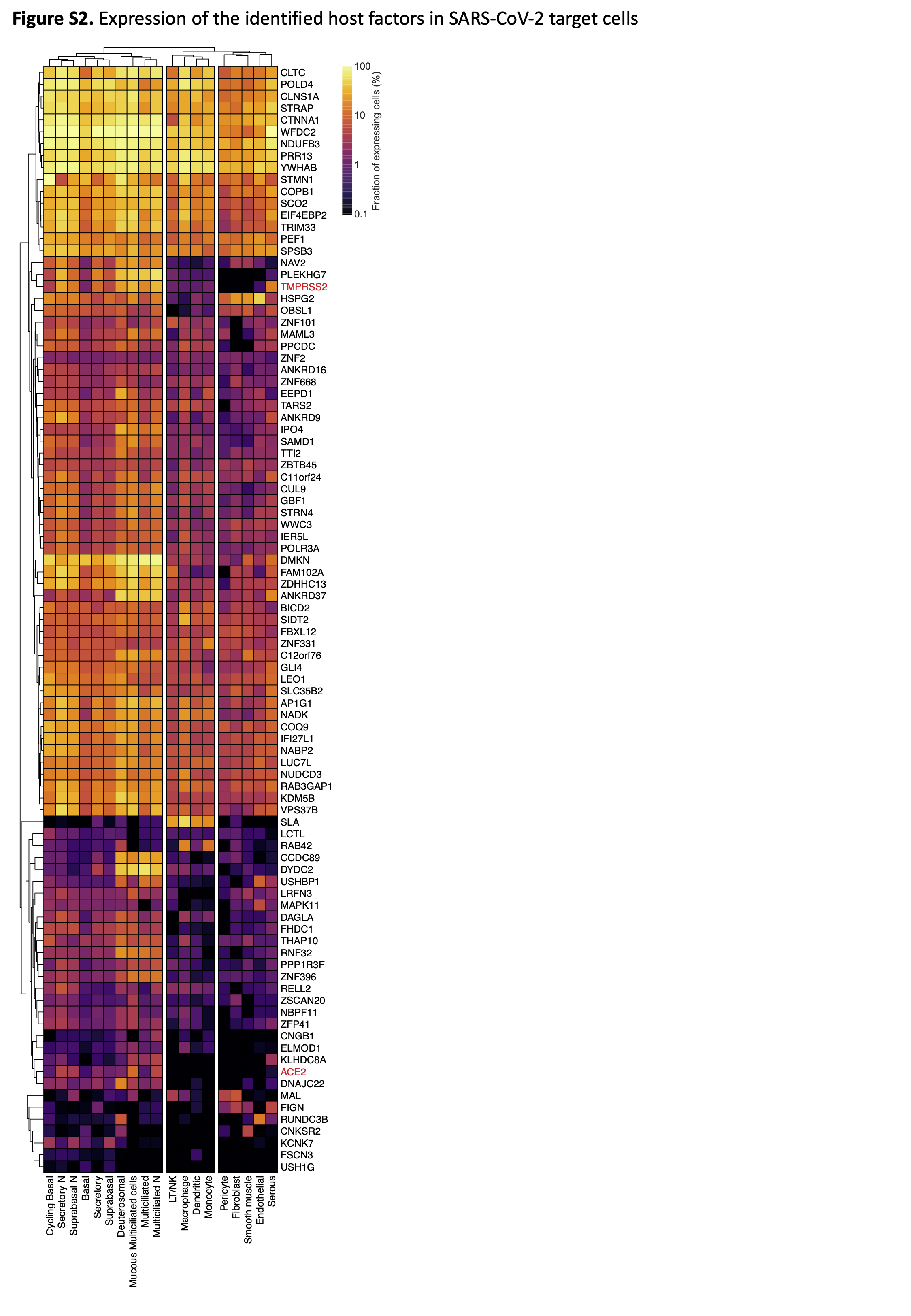

Supplement: S2 Fig — Heatmap shows percentage of detectable levels of expression of a given factor in the indicated cell type [74]. % expression >1 was considered a detectable level. The data underlying this figure can be found in S1 Data. (TIFF) [file pbio.3002738.s002.tiff]

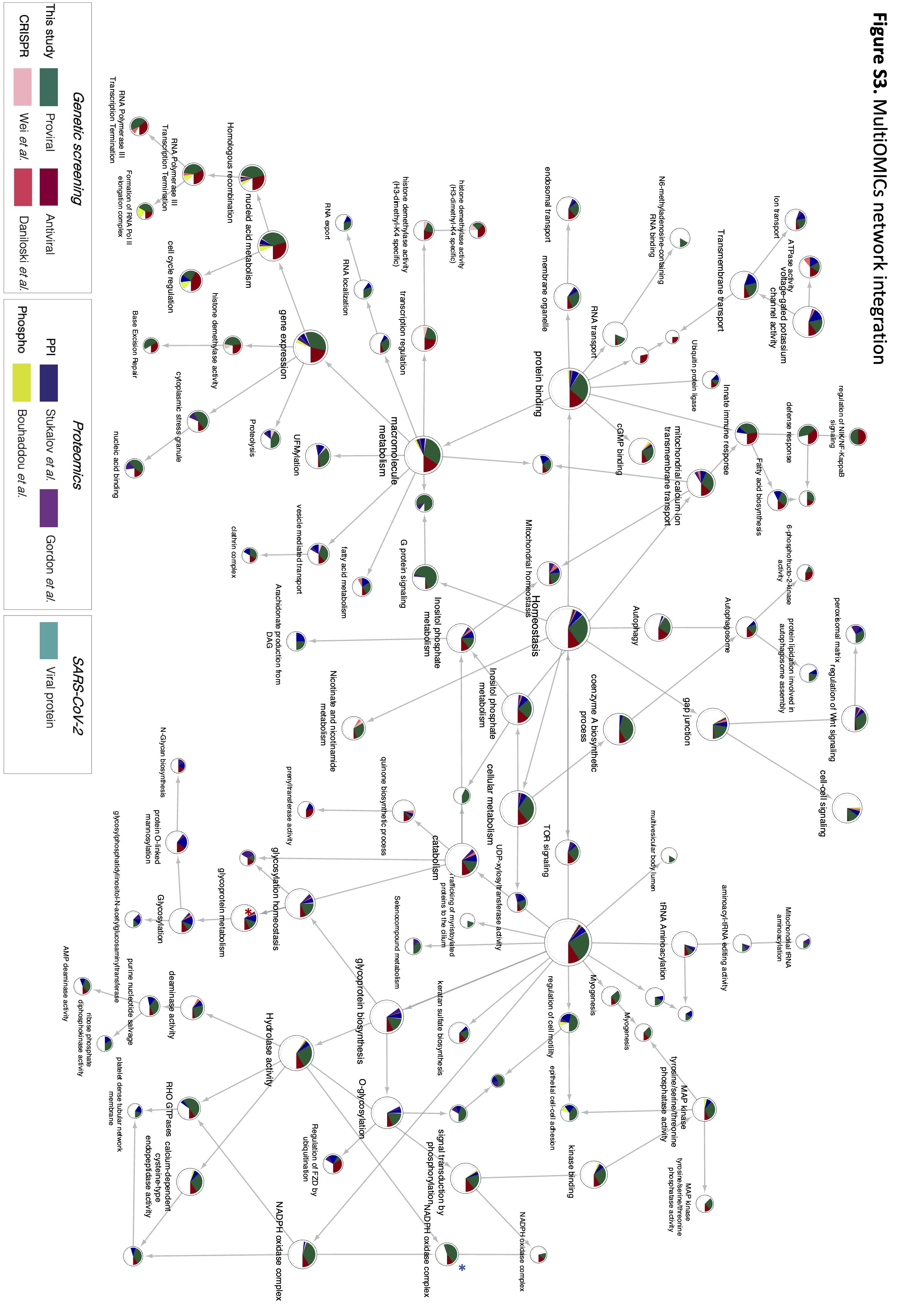

Supplement: S3 Fig — The network containing the identified proviral (green) and antiviral (red) human host factors was integrated with host factors reported to be relevant for SARS-CoV-2 infection. These include genetic CRISPR screen hits (Wei and colleagues, 2020 [15], light pink; Daniloski and colleagues, 2020 [16], dark pink), protein–protein interaction hits (Stukalov and colleagues, 2020 [31], blue; Gordon and colleagues, 2020 [32], purple), as well as hits from a phosphoproteomics study (Bouhaddou and colleagues, 2020 [33], yellow). The network was subjected to supervised community detection [67,73], and the resultant hierarchy is shown. Each node represents a cluster of densely interconnected proteins, and each edge (arrow) denotes containment of one community (edge target) by another (edge source). Labels indicate enriched biological processes. The percentage of each community that corresponds to each dataset is shown by matching colors. Edges indicate interactions from STRING database. * indicates highlighted clusters shown in Fig 2. (TIFF) [file pbio.3002738.s003.tiff]

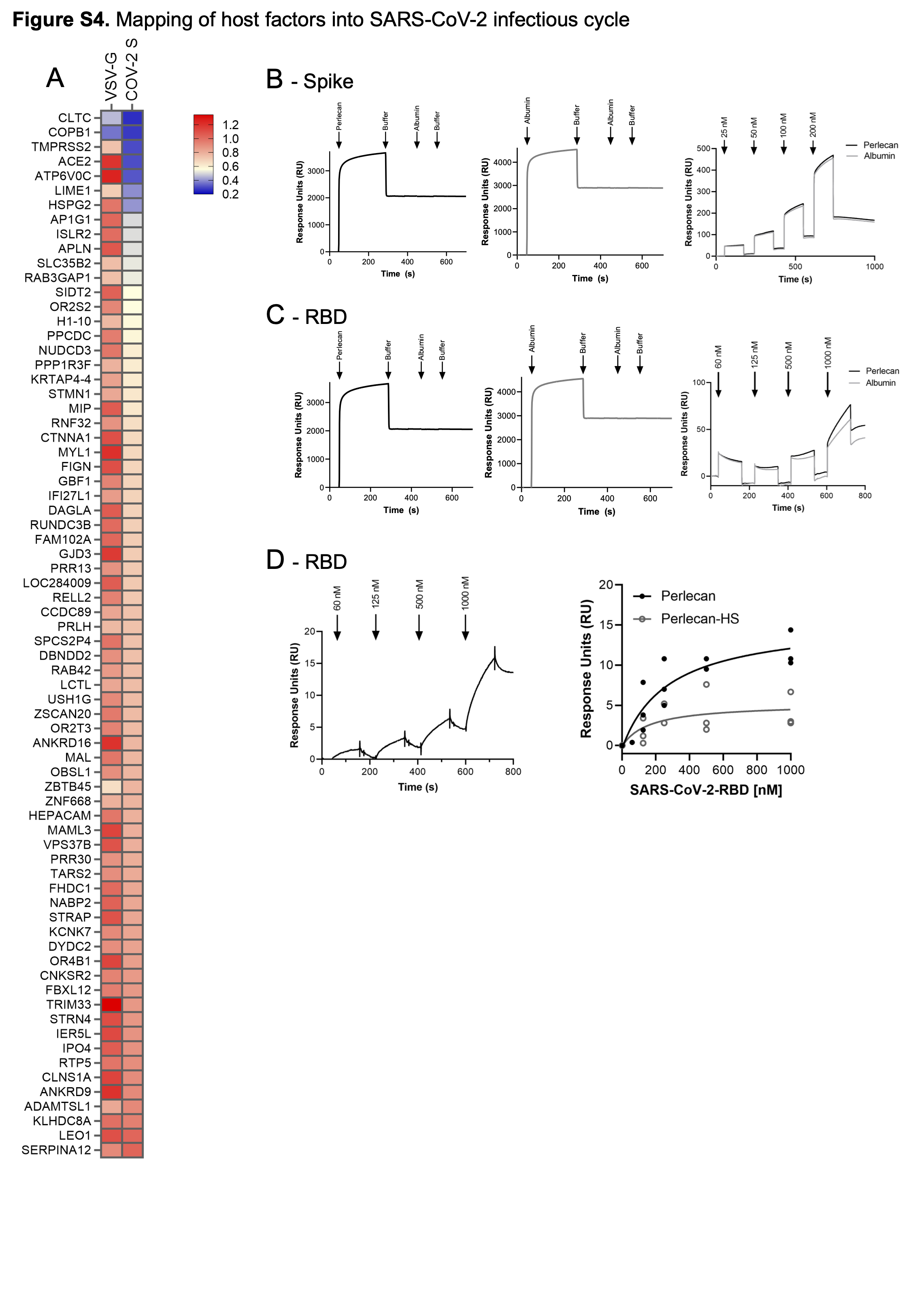

Supplement: S4 Fig — (A) Caco-2 cells subjected to siRNA-mediated knockdown of the indicated host factors were infected with SARS-CoV-2 pseudotyped VSV luciferase virus (VSV-S) or VSV luciferase virus expressing its natural glycoprotein (VSV-G) for 18h prior to measurement of luciferase signal. Data represent mean from one representative experiment in duplicate (n = 2). (B,C) Binding of spike protein and RBD to perlecan. Surface plasmon resonance (SPR) was used to evaluate spike binding to perlecan. This experiment was repeated twice. (D) Surface plasmon resonance (SPR) was used to evaluate binding of RBD to perlecan or perlecan without HS spike binding to immunopurified perlecan isolated from human coronary artery endothelial cells. Control flow channels contained immobilized BSA. RBD at indicated concentrations was run across the flow channels for 120 s and dissociation was measured in the following 600 s. The RU values throughout the experiment for BSA were subtracted from the RU values for perlecan to determine the level of specific binding. This experiment was repeated with perlecan treated with heparinase III. The data underlying this figure can be found in S1 Data. (TIFF) [file pbio.3002738.s004.tiff]

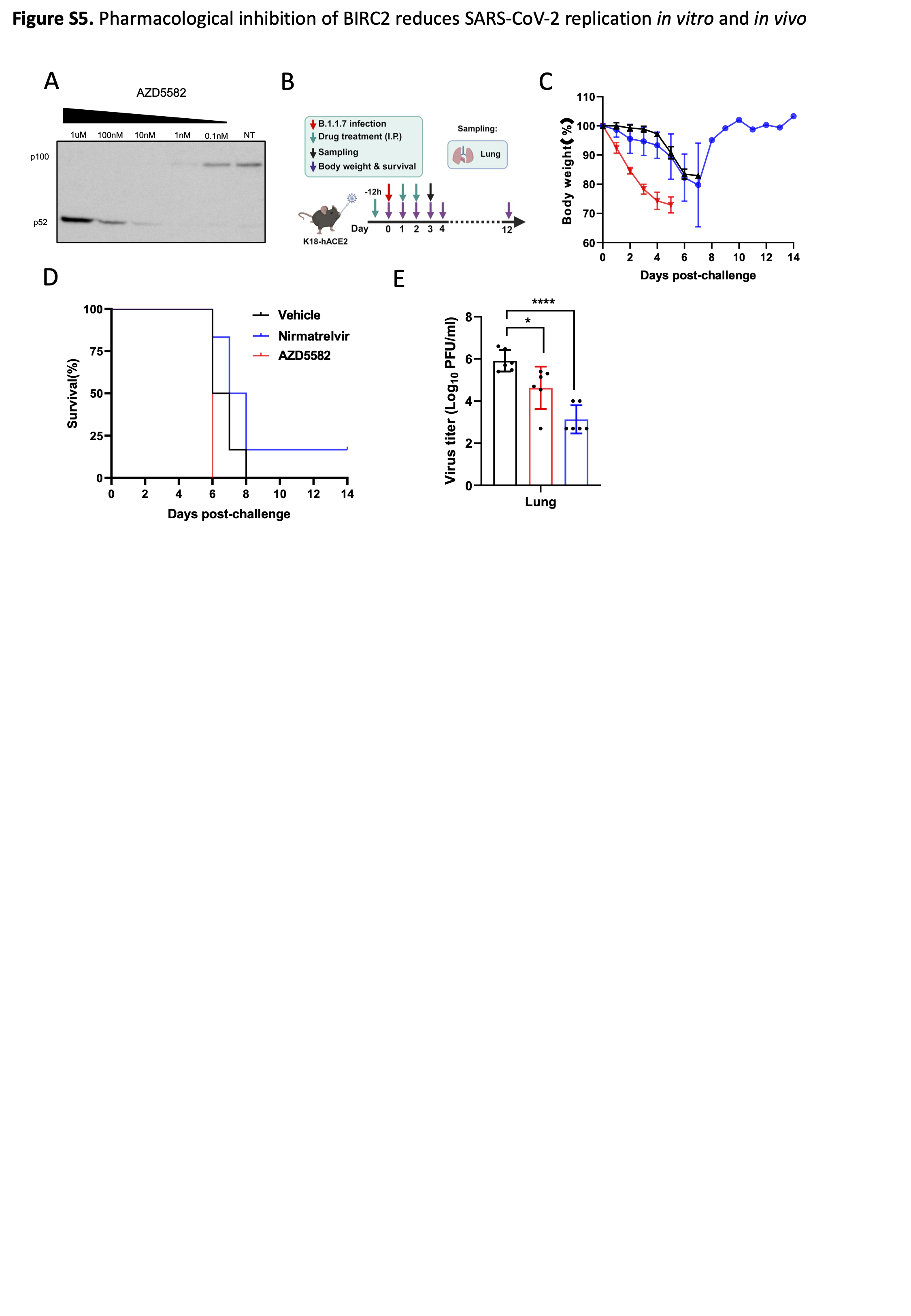

Supplement: S5 Fig — (A) Cells were treated with AZD5582 at the indicated concentrations. Twenty-four hours post-treatment, the cell lysates were analyzed by Western blotting for p100/p52 protein. A representative immunoblot presented here demonstrate that AZD5582 treatment induces the cleavage of p100. (B) Layout of mice experiments using SARS-CoV-2 B.1.1.7 (Alpha) infection. Effect of AZD5582 on SARS-CoV-2 replication in survival (C) and body weight (D) were recorded for 14 days post-infection. Virus titer as measured in the lungs of infected mice by plaque assay (E) were performed on 3 dpi. Tissue sampling was done at 72 hpi. One-way ANOVA when compared with the vehicle control group. *P < 0.05, ****P < 0.001. The data underlying this figure can be found in S1 Data. The original uncropped blots can be found in S1 Raw Images. (TIFF) [file pbio.3002738.s005.tiff]

Figure 4B

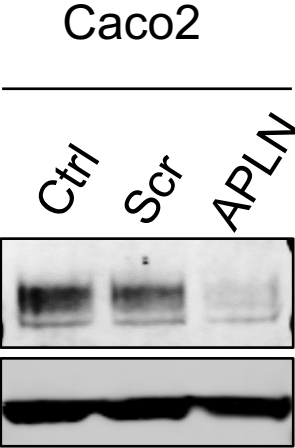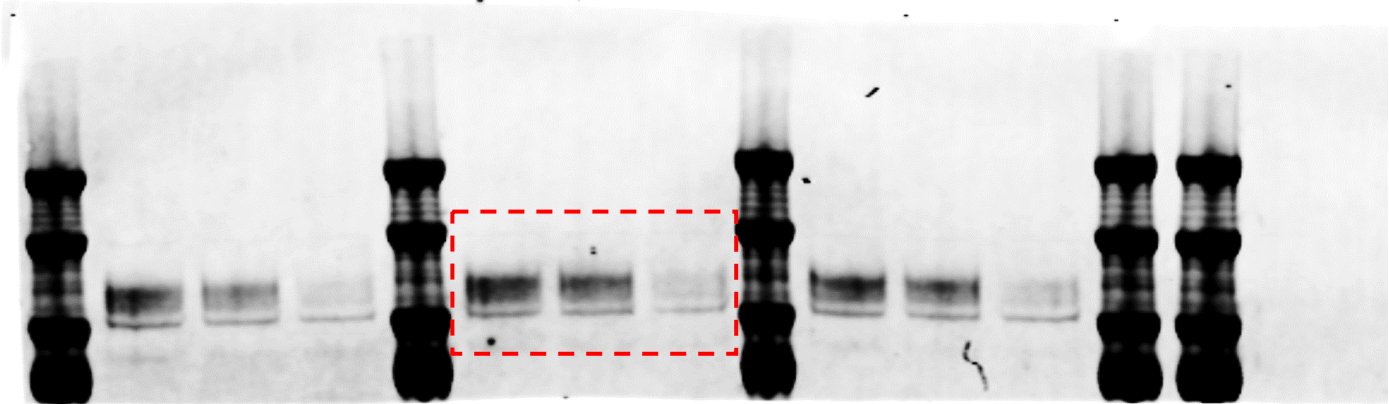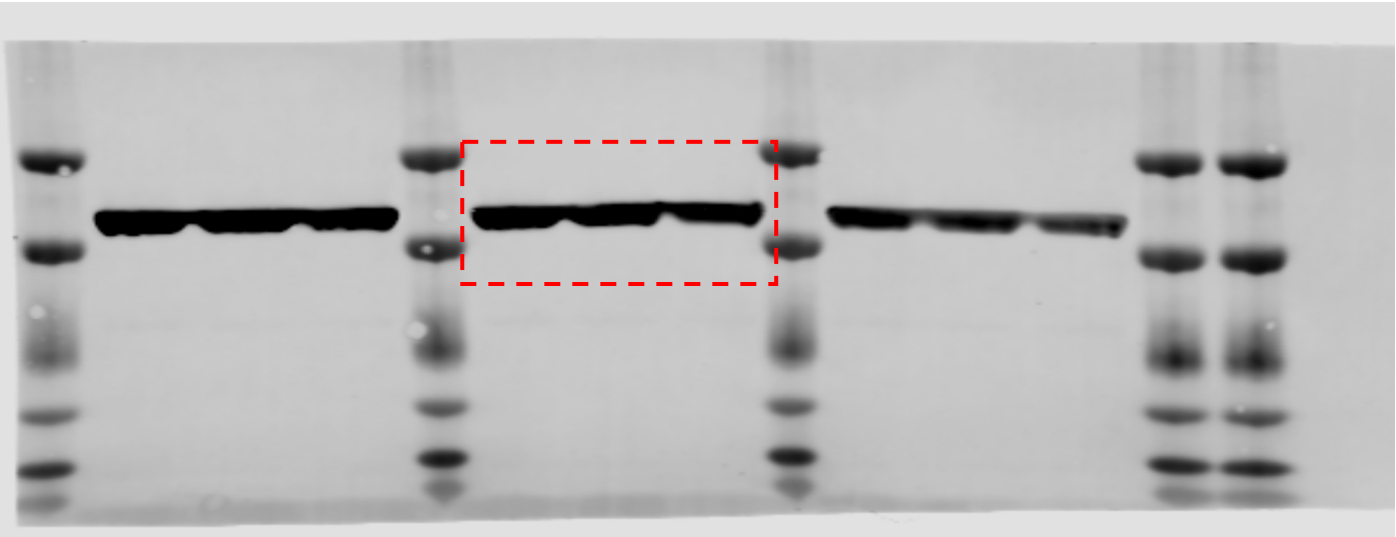

Figure S5A

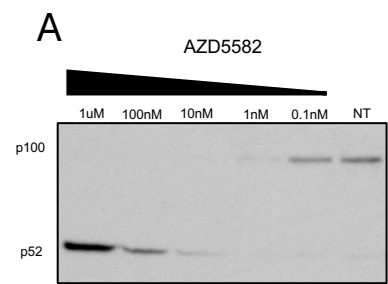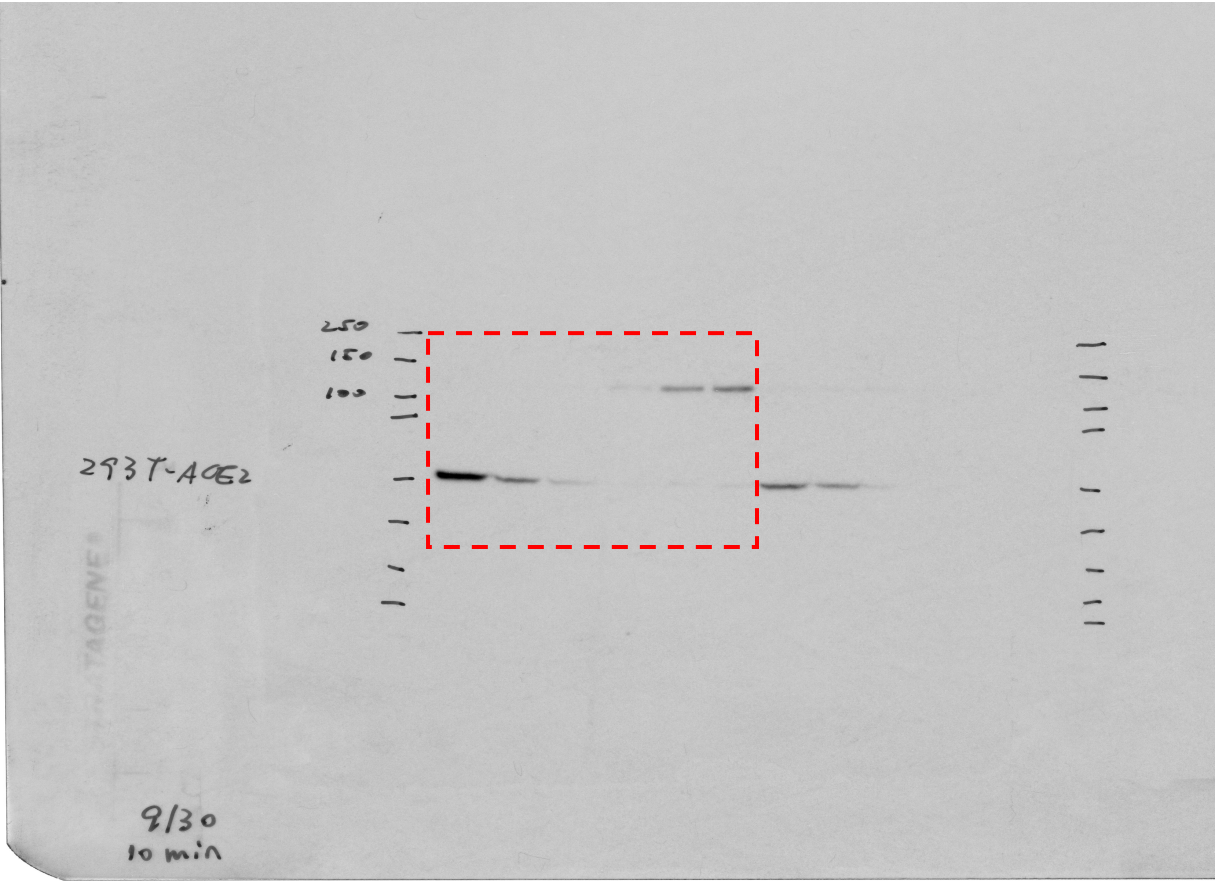

Supplement: S1 Raw Images — Uncropped western blots related to Figs 4B and S5A. (PDF) [file pbio.3002738.s010.pdf]
